# Ana1 helps recruit Polo to centrioles to promote mitotic PCM assembly and centriole elongation

**DOI:** 10.1242/jcs.258987

**Published:** 2021-07-22

**Authors:** Ines Alvarez-Rodrigo, Alan Wainman, Saroj Saurya, Jordan W. Raff

**Affiliations:** The Sir William Dunn School of Pathology, University of Oxford, South Parks Road, Oxford OX1 3RE, UK

**Keywords:** Polo, PLK1, Centrosome, Centriole, Pericentriolar material, PCM, Ana1, Cep295

## Abstract

Polo kinase (PLK1 in mammals) is a master cell cycle regulator that is recruited to various subcellular structures, often by its polo-box domain (PBD), which binds to phosphorylated S-pS/pT motifs. Polo/PLK1 kinases have multiple functions at centrioles and centrosomes, and we have previously shown that in *Drosophila* phosphorylated Sas-4 initiates Polo recruitment to newly formed centrioles, while phosphorylated Spd-2 recruits Polo to the pericentriolar material (PCM) that assembles around mother centrioles in mitosis. Here, we show that Ana1 (Cep295 in humans) also helps to recruit Polo to mother centrioles in *Drosophila*. If Ana1-dependent Polo recruitment is impaired, mother centrioles can still duplicate, disengage from their daughters and form functional cilia, but they can no longer efficiently assemble mitotic PCM or elongate during G2. We conclude that Ana1 helps recruit Polo to mother centrioles to specifically promote mitotic centrosome assembly and centriole elongation in G2, but not centriole duplication, centriole disengagement or cilia assembly.

This article has an associated First Person interview with the first author of the paper.

## Abstract (precis)

**Summary:** The C-terminus of Ana1 helps recruit Polo kinase to centrioles. This is key for efficient PCM assembly and centriole elongation, and independent from other roles of Ana1 in centriole maturation.

## INTRODUCTION

Polo kinase (PLK1 in mammals) is an important cell cycle regulator (Pintard and Archambault, 2018). During mitosis, it is recruited to several locations in the cell – such as centrosomes, kinetochores and the cytokinesis apparatus – where it performs multiple functions (Colicino and Hehnly, 2018). PLK1 is usually recruited to these locations by its polo-box domain (PBD) (Lee et al., 1998; Liu et al., 2004; Reynolds and Ohkura, 2003), which binds to phosphorylated S-pS/pT motifs in target proteins (Elia et al., 2003a,b). Mutating the first serine in the PBD-binding motif to threonine strongly reduces PBD binding *in vitro* and *in vivo* (Elia et al., 2003a,b; Decker et al., 2011; Joukov et al., 2014; Meng et al., 2015).

PLK1 has several key functions at centrosomes. These organelles are important microtubule (MT) organising centres that form around a pair of centrioles (comprising a mother and daughter centriole) when the mother recruits a matrix of pericentriolar material (PCM) around itself (Conduit et al., 2015b). During interphase, centrosomes organise relatively little PCM, but as cells prepare to enter mitosis the PCM expands dramatically in a process termed centrosome maturation (Palazzo et al., 1999). PLK1 is an essential driver of this process (Lane and Nigg, 1996; Haren et al., 2009), and several PCM proteins have been identified as PLK1 targets. In vertebrate cells, PLK1 phosphorylates pericentrin, which cooperates with CDK5RAP2 (also known as Cep215) to promote mitotic PCM assembly (Lee and Rhee, 2011; Kim and Rhee, 2014), whereas in flies and worms Polo/PLK1 kinases phosphorylate Cnn and SPD-5 (functional homologues of CDK5RAP2), respectively, which allows these proteins to assemble into a PCM scaffold around the mother centriole that recruits other PCM proteins (Conduit et al., 2014a,b; Woodruff et al., 2015; Wueseke et al., 2016; Feng et al., 2017).

Towards the end of mitosis, the mother and daughter centrioles disengage from each other. PLK1 is essential for disengagement (Tsou et al., 2009; Kim et al., 2015) and also for the subsequent maturation of the daughter centriole into a new mother centriole that is itself capable of duplicating and organising PCM (Loncarek et al., 2010; Wang et al., 2011; Kong et al., 2014; Shukla et al., 2015; Novak et al., 2016). The old mother (OM) and new mother (NM) centrioles then both duplicate during S phase by nucleating the assembly of a daughter centriole on their side. PLK1 is not essential for centriole duplication per se, but it is required for the growth of the centriole MTs that occurs during G2, at least in human cells (Kong et al., 2020), and for the subsequent maturation of the daughter centriole into a new mother centriole (Novak et al., 2016; Kong et al., 2014; Wang et al., 2011). After duplication in S phase, the two centrosomes (each now comprising a duplicated centriole pair) are held together by a linker, and PLK1 also helps disassemble this linker to promote centrosome separation as cells prepare to enter mitosis (Bertran et al., 2011; Mardin et al., 2011; Smith et al., 2011).

How PLK1 is recruited to centrosomes to execute its multiple functions is largely unclear, although this recruitment appears to be dependent on the PBD (Elia et al., 2003a,b; Hanisch et al., 2006; Jang et al., 2002; Lee et al., 1998; Seong et al., 2002; Song et al., 2000; Reynolds and Ohkura, 2003). In vertebrate systems, Cep192 is required for centrosome maturation (Gomez-Ferreria et al., 2007; Zhu et al., 2008) and it is phosphorylated by Aurora A (also known as AURKA) to create PBD-binding sites that recruit PLK1; this promotes the activation of both kinases at the centrosome (Joukov et al., 2010, 2014; Meng et al., 2015). The fly and worm homologues of Cep192, Spd-2 and SPD-2, respectively, are concentrated at centrioles and centrosomes, and their phosphorylation also helps recruit Polo/PLK1 kinases to the mitotic PCM to phosphorylate Cnn in flies and SPD-5 in worms (Decker et al., 2011; Alvarez-Rodrigo et al., 2019). In fly embryos, Spd-2, Polo and Cnn have been proposed to form a positive feedback loop that drives the expansion of the mitotic PCM around the mother centriole (Conduit et al., 2014b; Alvarez-Rodrigo et al., 2019). In this scenario, Spd-2 starts to be phosphorylated at centrioles as cells prepare to enter mitosis, and this allows Spd-2 to form a scaffold that can recruit other PCM proteins and that fluxes outwards from the mother centriole (Conduit et al., 2014b). The Spd-2 scaffold itself is weak, but it can recruit Polo and Cnn; the recruited Polo phosphorylates Cnn, which then forms a Cnn scaffold that recruits other PCM components and strengthens the Spd-2 scaffold (Conduit et al., 2014a). This allows more Spd-2 to accumulate around the centriole, which in turn drives the recruitment of more Polo and Cnn – so forming a positive feedback loop. In this way, Spd-2 recruits Polo and Cnn to the PCM to help drive centrosome maturation in flies.

If *Drosophila* Spd-2 cannot efficiently recruit Polo – because all its S-S/T motifs have been mutated to T-S/T – Polo recruitment to the PCM is dramatically reduced, but Polo is still strongly recruited to the mother centriole, indicating that other proteins must help recruit Polo to centrioles (Alvarez-Rodrigo et al., 2019). The centriole protein Sas-4 is phosphorylated by Cdk1 during mitosis on threonine 200 (T200), creating a PBD-binding site that recruits Polo to newly formed daughter centrioles (Novak et al., 2016). This allows the daughter to recruit Asl (Cep152 in humans), which allows the daughter to mature into a new mother that can duplicate and organise PCM – as Asl is required for both of these processes (Novak et al., 2014; Cizmecioglu et al., 2010; Dzhindzhev et al., 2010; Hatch et al., 2010; Conduit et al., 2014b). Although the single PBD-binding site in Sas-4 recruits Polo to mother centrioles, we suspected that other proteins must also be required. Here, we attempted to identify such proteins by mutating all the S-S/T motifs to T-S/T in several candidates. We show that the centriole protein Ana1 (Cep295 in humans) normally helps recruit Polo to mother centrioles. Ana1 and Cep295 are required for centriole maturation (Izquierdo et al., 2014; Fu et al., 2016; Tsuchiya et al., 2016), and in flies Ana1 helps recruit and/or maintain Asl at new mother centrioles (Fu et al., 2016; Saurya et al., 2016). Thus, flies lacking Ana1 lack centrioles, centrosomes and cilia (Blachon et al., 2009; Fu et al., 2016; Saurya et al., 2016), presumably because the centrioles cannot duplicate without Ana1 as they cannot recruit Asl. We show that centrioles that do not efficiently recruit Polo via Ana1 can still recruit Sas-4, Cep135 and Asl, and can still duplicate, disengage and organise cilia, but they cannot efficiently recruit mitotic PCM or elongate during G2. We propose that Ana1 recruits Polo to centrioles specifically to promote centriole elongation in G2 and mitotic PCM assembly.

## RESULTS

### A candidate screen for centriole proteins that help to recruit Polo to centrioles

**Fig. 1.**
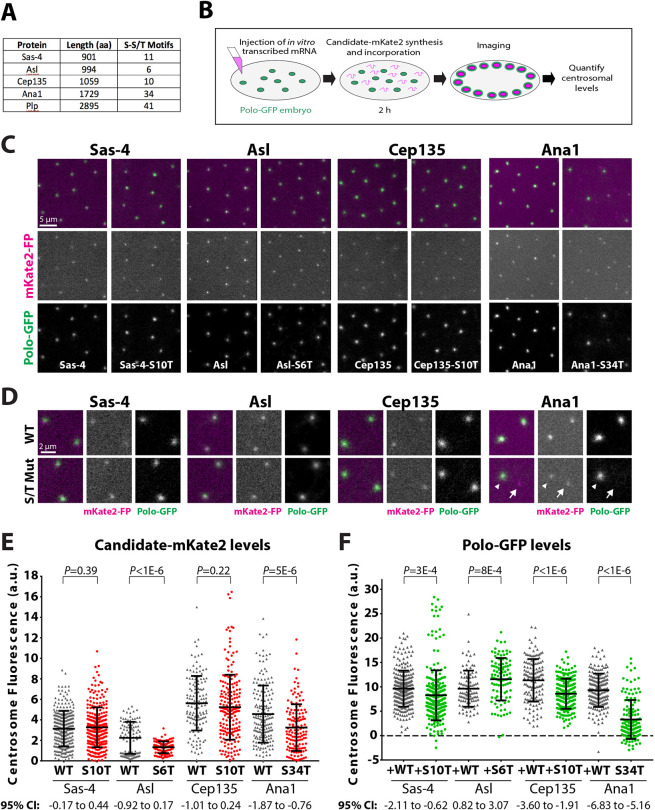
**An mRNA injection-based screen for proteins that help recruit Polo to centrosomes.** (A) Table shows the number of potential PBD-binding sites (S-S/T motifs) in several centriole proteins (aa, amino acids). (B) Schematic illustrates the mRNA injection assay used to test the effect on Polo recruitment of mutating all the potential PBD-binding sites in a candidate protein. Green circles represent centrosomes recruiting Polo–GFP. (C) Micrographs of embryos expressing Polo–GFP (green in merged channel, top) injected with mRNA encoding WT (left panels) or S*n*T mutant (right panels) forms of each of the candidate proteins (Sas-4, Asl, Cep135 or Ana1) tagged with mKate2 (mKate2–FP; magenta in merged channel, top). (D) Magnified view highlighting a pair of centrosomes for each condition, as described in C. Arrowheads indicate centrosomes that contain Ana1-S34T–mKate2 and that recruit Polo–GFP; arrows indicate centrosomes that contain Ana1-S34T–mKate2 but do not detectably recruit Polo–GFP. A total of 5–14 embryos were injected and analysed for each mRNA. Note that Ana1 is normally significantly brighter at OM centrioles than at NM centrioles (Saurya et al., 2016) (Fig. S1B,C), making it hard to visually infer the relative amount of fluorescent fusion protein at OM and NM centrioles in these mRNA injection experiments. (E,F) Graphs quantify (E) the centrosomal levels of either the WT or mutant candidate–mKate2 fusions and (F) the corresponding centrosomal levels of Polo–GFP in each condition in S phase (a.u. arbitrary units). A total of 8–10 pairs of centrosomes were analysed per embryo (*n*=276, 280, 100, 100, 140, 198, 180 and 140 for WT and S*n*T Sas-4, Asl, Cep135 and Ana1, respectively). Note that the distribution of WT Asl is bimodal as Asl is usually much brighter at OM centrioles than NM centrioles (Novak et al., 2014); this effect is less pronounced in the Asl-S6T mutant for unknown reasons. Error bars represent s.d. *P*-values were calculated using an unpaired two-tailed *t*-test with Welch's correction.

To identify proteins involved in recruiting Polo to the mother centriole we examined a small number of candidates that are important for centriole assembly and/or function in flies and that, like Polo, localise in a ring around the mother centriole: Sas-4 (known as CPAP in vertebrates), Asl, Cep135, Ana1 and PLP (the *Drosophila* homologue of pericentrin) (Mennella et al., 2012; Fu and Glover, 2012; Fu et al., 2016; Saurya et al., 2016; Tian et al., 2021). The PBD is required to efficiently target PLK1 to centrioles and centrosomes (Elia et al., 2003a,b; Hanisch et al., 2006; Jang et al., 2002; Lee et al., 1998; Seong et al., 2002; Song et al., 2000; Reynolds and Ohkura, 2003), and we previously uncovered the role of Spd-2 in recruiting Polo to the mitotic PCM by mutating all 34 of the potential PBD-binding S-S/T motifs in Spd-2 to T-S/T. This S-to-T substitution is conservative (French and Robson, 1983), so is unlikely to dramatically perturb protein structure, but it abolishes PBD binding *in vitro* and *in vivo* (Elia et al., 2003a,b; Hanisch et al., 2006; Jang et al., 2002; Lee et al., 1998; Seong et al., 2002; Song et al., 2000; Reynolds and Ohkura, 2003). We generated mutant versions of all the candidate proteins in which we mutated all S-S/T motifs to T-S/T (Fig. 1A). The only exception was Sas-4, for which all S-S/T motifs except the previously identified T200 S-T motif – which has already been shown to initiate Polo recruitment at centrioles (Novak et al., 2016) – were mutated. We then analysed the centrosomal recruitment of each protein and of Polo–GFP using an mRNA injection strategy, where proteins encoded by an injected mRNA are gradually translated and so eventually outcompete the endogenous (unlabelled) protein for binding to the centriole (Fig. 1B) (Novak et al., 2016; Alvarez-Rodrigo et al., 2019).

We produced mRNA *in vitro* encoding either wild-type (WT) or the S-to-T substitution (S*n*T, where *n* indicates the number of substitutions) versions of the candidate proteins followed by a C-terminal red fluorescent tag (mKate2), and injected this into embryos expressing Polo–GFP (Buszczak et al., 2007). The embryos were imaged 2 h after injection to allow the injected mRNA to be translated, the fluorescent tag to mature and the protein to incorporate into centrosomes. Unfortunately, neither the WT-PLP–mKate2 nor PLP-S41T–mKate2 fusion proteins were detectable at centrosomes in these experiments, probably because PLP is so large that more time is required for the protein to be translated and for the fluorophore to mature. PLP was therefore excluded from further analyses. All other candidate proteins were detectable at centrosomes, and the WT and S*n*T-mutant proteins exhibited qualitatively similar localisations (Fig. 1C,D). The levels of protein expression induced in these mRNA injection experiments was variable from embryo to embryo, but the average centriolar fluorescence intensity of the WT and S*n*T mutants was similar for Sas-4 and Cep135, and was slightly reduced for Ana1-S34T (∼30%) and Asl-S6T (∼40%) compared to their WT proteins (Fig. 1E).

For Asl, Cep135 and Sas-4, the recruitment of Polo–GFP to centrosomes was qualitatively similar in embryos expressing either the WT or S*n*T-mutant forms of the protein (Fig. 1C,D), and on average ∼20% more and ∼15% and ∼25% less Polo–GFP, respectively, was recruited to centrioles in the presence of the mutant proteins compared to their WT counterparts (Fig. 1F). As we do not know the relative expression levels of these fusion proteins compared to their endogenous counterparts, we are cautious in interpreting these findings. Nevertheless, these differences in Polo recruitment are relatively small compared to those we have observed in other cases where we have mutated S-S/T motifs (Novak et al., 2016; Alvarez-Rodrigo et al., 2019). We therefore tentatively conclude that none of the S-S/T motifs in Asl, Cep135 or Sas-4 that we tested here play a major part in recruiting Polo to centrioles in flies.

### Mutation of the potential PBD-binding sites in Ana1 dramatically reduces centrosomal Polo levels

In contrast, an average of ∼65% less Polo–GFP was recruited to centrioles in the presence of Ana1-S34T compared to the WT protein, and this reduction was highly asymmetric, with one centriole in a separating centriole pair usually exhibiting normal or only slightly reduced levels of Polo–GFP and the other exhibiting a severe reduction (Fig. 1). This striking asymmetry was observed in 7/7 embryos injected with Ana1-S34T–mKate2 mRNA and 0/9 embryos injected with WT-Ana1–mKate2 mRNA (scored blind). Interestingly, a very similar asymmetric loss of Polo–GFP was observed with the Sas-4-T200 mutant protein, where it was shown that it was always the NM centriole that did not properly recruit Polo (Novak et al., 2016). This asymmetric behaviour is likely a consequence of the significant fraction of Ana1 and Sas-4 that incorporate into assembling centrioles irreversibly (Saurya et al., 2016; Conduit et al., 2015a). As a result, OM centrioles will tend to have incorporated less mutant protein in these experiments, as they were formed earlier in development when less mutant protein (translated from the injected mRNA) was available.

### Ana1-S34T fusion proteins support centriole and cilium assembly

**Fig. 2.**
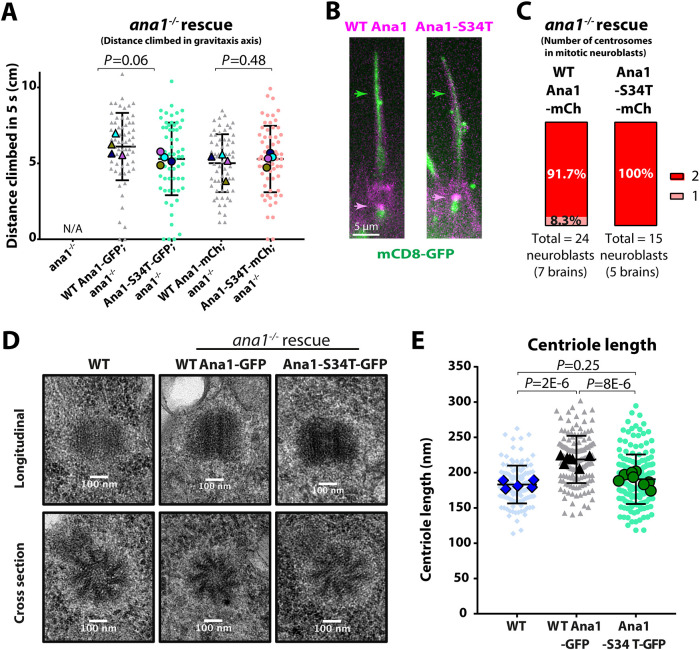
**The Ana1-S34T protein appears to be largely functional.** (A) Graphs show the quantification of negative gravitaxis climbing assays. Each small shape shows the distance climbed by one of 15 individual *ana1^−/−^* flies expressing either WT or S34T-mutant versions of Ana1 tagged with either GFP or mCherry (mCh) after being tapped to the bottom of a cylinder. The larger shapes show the average distance climbed by all flies in four technical repeats. Note that *ana1^−/−^* flies without any transgene were not scored in this assay, as all of the mutant flies were severely uncoordinated due to the lack of cilia and so did not climb at all. Nevertheless, we show this bar as zero – marked with not applicable (N/A) – to better illustrate the level of rescue for each transgene. (B) Micrographs show the cilia membrane (green arrow) in a sensory neuron marked with mCD8–GFP (green) extending into a sensory bristle in an antenna from an adult *ana1^−/−^* mutant fly expressing either WT-Ana1–mCherry or Ana1-S34T–mCherry (magenta), which both localise to the cilium basal body (magenta arrow). We examined >20 bristles in seven antennae from four different females (>100 in total) and detected no obvious morphological differences between the WT and mutant conditions. (C) Quantification of the percentage of mitotic neuroblasts with one or two centrosomes in *ana1^−/−^* larval brains co-expressing Spd-2–GFP and either WT-Ana1–mCherry or Ana1-S34T–mCherry. Live neuroblasts were analysed blind, with centrosomes being identified by the colocalisation of both markers. (D) Electron micrographs show longitudinal (top) and cross-section (bottom) views of typical centrioles in either WT or *ana1^−/−^* mutant third-instar larval wing-disc cells expressing either WT-Ana1–GFP or Ana1-S34T–GFP. (E) Graph quantifies the average longitudinal length of the centrioles in each condition (scored blind). As shown previously (Saurya et al., 2016), centrioles are slightly elongated when WT-Ana1–GFP is overexpressed, but this was not the case when Ana1-S34T–GFP was overexpressed. Small shapes indicate individual centriole lengths (126, 142 and 172, respectively), large shapes indicate the average centriole length in a whole wing disc (*n*=5, 7 and 8, respectively). Error bars represent s.d. *P*-values in A and E were calculated using an unpaired two-tailed *t*-test with Welch's correction and the ordinary one-way ANOVA with Tukey's multiple comparison test, respectively.

Although these mRNA injection experiments indicate that the Ana1-S34T protein inhibits Polo recruitment to centrioles and centrosomes, we wondered whether this might be because it disrupts centriole structure and/or function more generally. We therefore generated transgenic fly lines expressing either WT-Ana1 or Ana1-S34T constructs with a C-terminal GFP or mCherry tag under the control of the *ubiquitin* promoter, which drives moderate expression in all tissues (Lee et al., 1988). As described above, *ana1^−/−^* mutant flies lack detectable centrioles, centrosomes and cilia, and they die shortly after eclosion (Blachon et al., 2009) because they are uncoordinated due to the lack of cilia in their sensory neurons (Kernan et al., 1994). Both the WT and mutant transgenes equally rescued the uncoordinated phenotype of *ana1^−/−^* mutants (Fig. 2A), and we could detect no morphological difference between the cilia in the sensory neurons of *ana1^−/−^* mutants expressing the two transgenes (Fig. 2B). Moreover, *ana1^−/−^* mutant third-instar larval neuroblasts – which normally lack detectable centrioles (Blachon et al., 2009) – exhibited normal numbers of centrioles when rescued by either the WT or mutant transgenes (Fig. 2C). Finally, we examined WT and S34T-mutant centrioles in larval wing discs using electron microscopy (EM), and the only difference we could detect was that, unlike the WT protein (Saurya et al., 2016), the mutant protein was unable to promote the slight over-elongation of the centrioles (Fig. 2D,E; all centrioles scored blind; see below). Taken together, these data indicate that the centrioles in cells expressing Ana1-S34T fusion proteins are not generally disorganised or perturbed, and that the mutant proteins can rescue the *ana1^−/−^* mutant phenotype by supporting accurate centriole duplication and cilium assembly.

### Embryos expressing Ana1-S34T transgenes die early in development

We noticed, however, that *ana1^−/−^* mutant females expressing Ana1-S34T–GFP were essentially sterile, laying embryos that hatched at a frequency of only ∼0.4% (*n*>1000) compared to ∼85% (*n*>500) for those laid by mutant females expressing WT-Ana1–GFP (and we obtained similar results with flies expressing Ana1–mCherry fusions). This difference was not due to differential expression, as the WT and mutant transgenes were expressed at similar levels in embryos (Fig. S1A), and centriolar levels of the mutant protein were actually slightly higher than the WT protein on both OM and NM centrioles (Fig. S1B,C).

Such female sterility is often associated with proteins required for efficient centrosome assembly in flies (e.g. Spd-2, Cnn and TACC) (Dix and Raff, 2007; Megraw et al., 1999; Gergely et al., 2000). This is because centrosomes are not essential for cell division in fly somatic cells (Basto et al., 2006), but are essential for the very rapid rounds of mitosis that occur in the developing syncytial embryo (Megraw et al., 1999; Varmark et al., 2007; Stevens et al., 2007). An analysis of fixed embryos laid by *ana1^−/−^* mutant females expressing Ana1-S34T–GFP confirmed that they largely died during the syncytial stages in a manner consistent with the gradual accumulation of centrosome and mitotic defects (Fig. S2). For simplicity, we hereafter refer to embryos laid by *ana1^−/−^* mutant females (embryos lacking any endogenous supply of WT untagged Ana1 protein) that express either a WT or mutant Ana1 fusion protein as WT-Ana1 or Ana1-S34T embryos, respectively.

### Ana1 helps to recruit Polo to the mother centriole

**Fig. 3.**
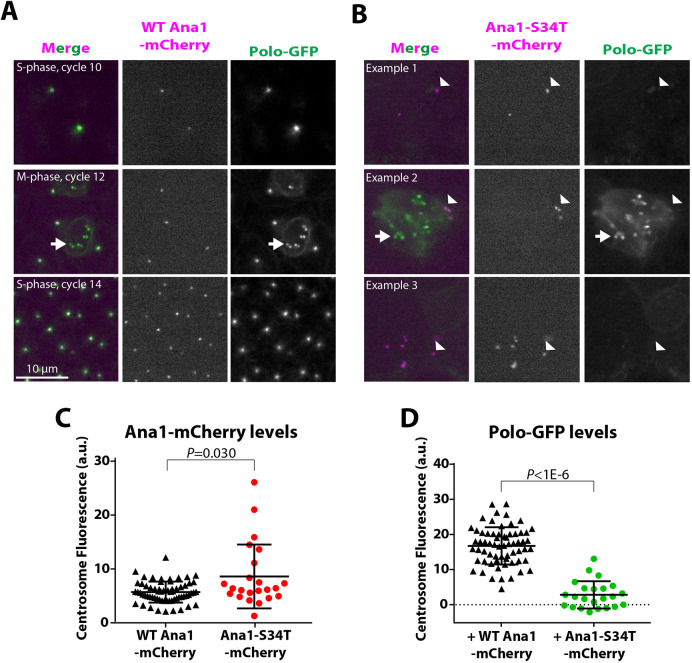
**Centrosomal Polo recruitment is severely perturbed in Ana1-S34T embryos.** (A,B) Examples of conventional spinning disk confocal images from living (A) WT-Ana1–mCherry and (B) Ana1-S34T–mCherry (magenta) embryos co-expressing Polo–GFP (green). Polo–GFP localised strongly to centrosomes at all stages of development and of the nuclear division cycle in WT-Ana1–mCherry embryos (A), also strongly staining the kinetochores during mitosis (arrows). Although embryos expressing Polo–GFP and Ana1-S34T–mCherry were very sick (and so difficult to accurately stage), Polo–GFP was usually barely detectable at the centrosomes (B, arrowheads), but was still strongly recruited to kinetochores (B, arrows) in the embryos that were in mitosis. (C,D) Graphs show the mean centrosomal (C) Ana1–mCherry and (D) Polo–GFP intensities in WT-Ana1–mCherry (black) or Ana1-S34T–mCherry embryos (red and green, respectively; a.u., arbitrary units). In total, *n*=64 centrosomes from ten different embryos co-expressing Polo–GFP and WT-Ana1–mCherry and *n*=23 centrosomes from six different embryos co-expressing Polo–GFP and Ana1-S34T–mCherry. Error bars indicate s.d. *P*-values were calculated using an unpaired two-tailed *t*-test with Welch's correction.

We reasoned that the centrioles in Ana1-S34T embryos might be unable to form fully functional centrosomes because they cannot recruit Polo efficiently (consistent with the results of our mRNA injection experiments). To test this possibility, we expressed Polo–GFP in embryos laid by *ana1^−/−^* mutant females that expressed either WT-Ana1–mCherry or Ana1-S34T–mCherry. The WT-Ana1 embryos expressing Polo–GFP developed normally, but Ana1-S34T embryos expressing Polo–GFP had mitotic defects that were much more severe than those observed in Ana1-S34T embryos alone, suggesting that the GFP-tagged Polo sensitises the embryos to the expression of Ana1-S34T (Fig. 3A,B). We observed a similar sensitisation when Polo–GFP was co-expressed with mutant forms of Spd-2 that were unable to recruit Polo to the mitotic PCM (Alvarez-Rodrigo et al., 2019). Centriolar levels of Ana1-S34T–mCherry and WT-Ana1–mCherry were similar in these embryos (Fig. 3C; Fig. S3A), but centrosomal Polo–GFP levels were dramatically reduced in the Ana1-S34T embryos (Fig. 3D; Fig. S3B).

**Fig. 4.**
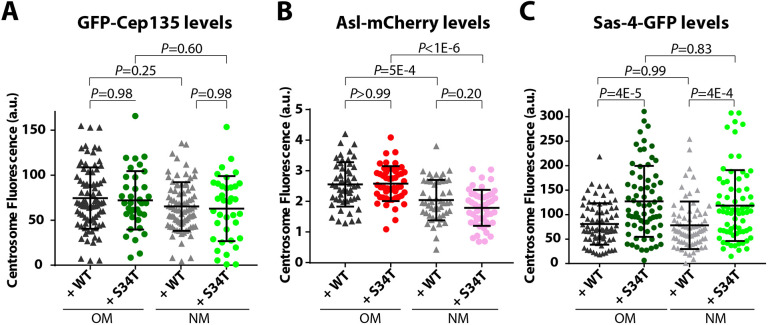
**The centriole recruitment of Cep135, Asl and Sas-4 is largely unperturbed in Ana1-S34T embryos.** (A) Graph shows the mean centrosomal GFP–Cep135 intensity in WT-Ana1–mCherry (+WT; black and grey triangles) or Ana1-S34T–mCherry (+S34T; green and light green circles) embryos (11 and seven embryos, respectively) in S phase. Multiple centrosome pairs were analysed per embryo (*n*=82 and 37 pairs in total for WT-Ana1 and Ana1-S34T embryos, respectively), for each pair the centrosomes were classified as OM or NM based on their Ana1–mCherry levels (data not shown). (B) Graph shows mean Asl–mCherry intensity at OM and NM centrosomes in WT-Ana1–GFP embryos (+WT; black and grey triangles) or Ana1-S34T–GFP embryos (+S34T; red and pink circles) in S phase. Five embryos were analysed per genotype, and ten pairs of centrosomes were analysed per embryo (*n*=50 centrosome pairs each). For each pair, the centrosome with the highest mean Asl–mCherry intensity was classified as the OM (Novak et al., 2014). (C) Graph shows mean centrosomal Sas-4–GFP intensity in WT-Ana1–mCherry embryos (+WT; black and grey triangles) or Ana1-S34T–mCherry embryos (+S34T; green and light green circles) at the beginning of S-phase. Eight embryos were analysed per genotype, and multiple centrosome pairs were analysed per embryo (*n*=74 and 70 pairs in total). For each pair the centrosomes were classified as OM or NM based on their Ana1–mCherry levels (data not shown). Error bars represent s.d. *P*-values were calculated using the ordinary one-way ANOVA with Tukey's multiple comparison test. a.u., arbitrary units.

Ana1 interacts with several other centriole assembly proteins, such as Cep135, Asl and Sas-4 (Fu et al., 2016; Galletta et al., 2016). We therefore tested whether the centriolar recruitment of any of these proteins was perturbed in Ana1-S34T embryos. The centriolar levels of GFP–Cep135 (Fig. 4A) and Asl–mCherry (Fig. 4B) were indistinguishable in WT-Ana1 and Ana1-S34T embryos, while the centriolar levels of Sas-4–GFP were slightly increased in the Ana1-S34T embryos (Fig. 4C). Thus, the centrioles in Ana1-S34T embryos can still recruit several key proteins, and the defect in Polo recruitment is not an indirect consequence of a failure to recruit and/or maintain Cep135, Asl or Sas-4 at centrioles.

**Fig. 5.**
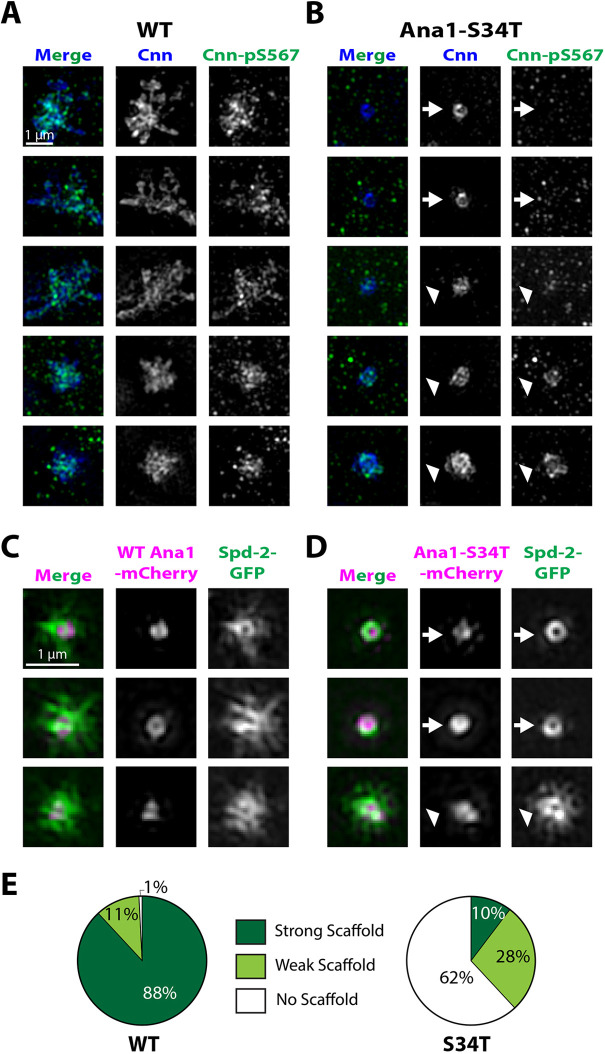
**Mitotic PCM expansion is impaired in Ana1-S34T embryos.** (A,B) 3D-SIM images from fixed (A) WT-Ana1–mCherry and (B) Ana1-S34T–mCherry embryos. The embryos were stained with a general Cnn antibody (blue) and an antibody that recognises a specific Polo-dependent phospho-epitope in Cnn (Cnn-pS567, green; Feng et al., 2017). Cnn phosphorylated at S567 was detected at high levels within the PCM in WT-Ana1–mCherry embryos (A), indicating that Polo is present within the PCM. In Ana1-S34T–mCherry embryos (B), Ser567 phosphorylated Cnn was present in some centrosomes (arrowheads), but not in others (arrows), even though unphosphorylated Cnn was present at the centriole wall in all cases. The lack of Cnn-pS567 was correlated with a lack of mitotic PCM expansion, suggesting that these centrioles lacked sufficient Polo to phosphorylate Cnn and to drive PCM expansion. (C,D) Micrographs show 3D-SIM images of individual centrosomes in living (C) WT-Ana1–mCherry or (D) Ana1-S34T–mCherry (magenta in merged images) embryos expressing Spd-2–GFP (green in merged images). While Ana1–mCherry images are shown here for reference, reliably reconstructing the Ana1–mCherry signal was challenging, due to its low levels and the fast bleaching of the fluorophore. Thus, images were selected for analysis based only on whether the Spd-2–GFP reconstructed image was deemed of sufficient quality by SIMcheck (Ball et al., 2015). All centrosomes were imaged in approximately mid-S-phase when the centrosomal levels of Spd-2 are maximal. All the centrosomes in WT-Ana1–mCherry embryos organised Spd-2–GFP PCM scaffolds, but this was true only in a minority of Ana1-S34T–mCherry embryos (arrowheads), where many centrosomes recruited Spd-2–GFP only to the centriole wall (arrows). (E) Pie charts quantify the percentage of centrosomes that qualitatively showed a strong (dark green), weak (light green) or no (white) pericentriolar scaffold (*n*=21 reconstructed centrioles for each genotype, scored blind).

We wanted to use 3D structured illumination super-resolution microscopy (3D-SIM) to test whether Ana1 helps recruit Polo to the centriole wall, the mitotic PCM, or both. Because the Ana1-S34T–mCherry embryos co-expressing Polo–GFP were very sick, we could not obtain images of sufficient quality to pass our usual SIMcheck quality control (Ball et al., 2015). Moreover, none of the commercially available anti-PLK1 antibodies that we tested detectably recognised Polo in fixed embryos, so we could not assess Polo localisation in embryos expressing only the endogenous Polo. We therefore used an antibody that recognises a phospho-epitope in Cnn (Cnn-pS567) as a proxy for centrosomal Polo activity, as this epitope is specifically phosphorylated at centrosomes by Polo (Feng et al., 2017; Alvarez-Rodrigo et al., 2019). The centrosomes in WT-Ana1–mCherry embryos invariably organised a robust Cnn scaffold that contained Cnn-pS567 (Fig. 5A). Strikingly, in Ana1-S34T–mCherry embryos, the centrosomes exhibited a clear heterogeneity. All of the centrosomes recruited a small amount of Cnn around the mother centriole, but most centrosomes were devoid of Cnn-pS567 (Fig. 5B, arrows), while others had detectable, but usually low, levels (Fig. 5B, arrowheads) – and these latter centrosomes were invariably associated with at least some Cnn scaffold that extended around the mother centriole.

### Polo recruitment by Ana1 is required for efficient PCM scaffold assembly

We observed a similar heterogeneity in PCM scaffold assembly in living WT-Ana1–mCherry or Ana1-S34T–mCherry embryos expressing Spd-2–GFP (Fig. 5C,D). Spd-2 was concentrated at the centriole wall of all the centrosomes in both WT-Ana1 and Ana1-S34T embryos, but while most of the centrosomes in the WT embryos also organised an extensive Spd-2 scaffold (Fig. 5C,E), only ∼10% of the centrosomes in the Ana1-S34T embryos did so (Fig. 5D, arrowheads; Fig. 5E), with most centrioles organising no detectable PCM scaffold (Fig. 5D, arrows; Fig. 5E) (all images scored blind). We conclude that the failure to recruit Polo to centrioles in Ana1-S34T embryos is not due to a failure to recruit Spd-2 to centrioles, as centriolar Spd-2 appears to be recruited normally in these embryos. Moreover, while PCM scaffold assembly is largely suppressed on most Ana1-S34T centrioles, a significant minority can organise at least some scaffold that contains Spd-2 and Cnn-pS567 and that extends outwards around the centrioles.

**Fig. 6.**
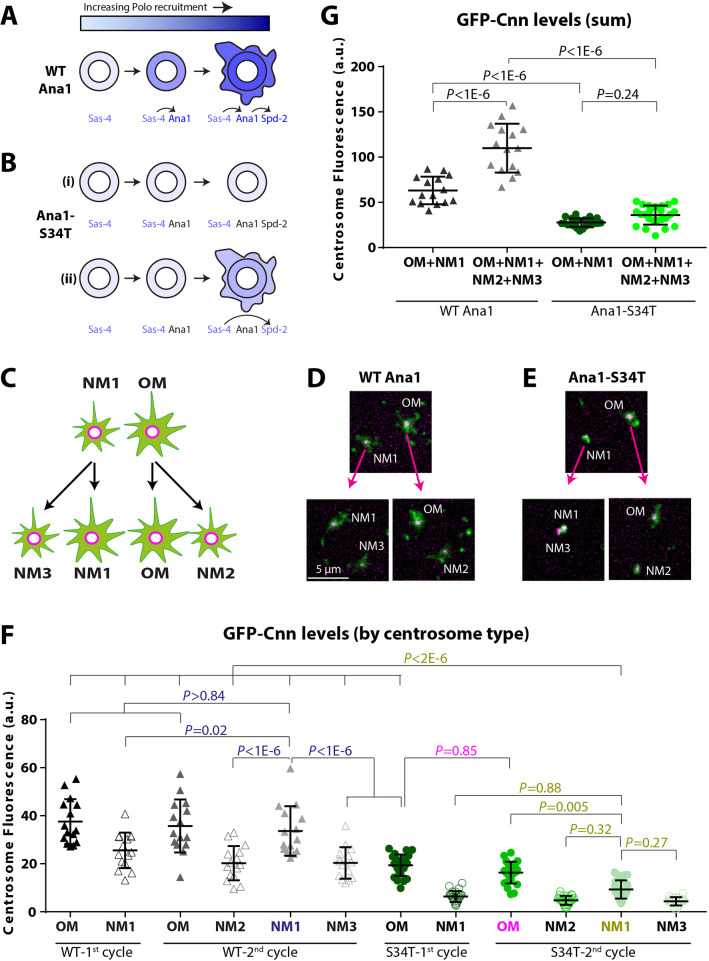
**Some OM centrioles can partially bypass the requirement for Ana1 to help recruit Polo to centrioles, and so recruit some of the mitotic scaffold protein Cnn.** (A,B) Schematic illustrates (A) how a sequential phosphorylation cascade comprising Sas-4, Ana1 and Spd-2 might drive increasing levels of Polo recruitment to the mother centriole and then to the expanding mitotic PCM in WT embryos, and (B) how this process might be perturbed in embryos expressing a form of Ana1 (Ana1-S34T) that cannot efficiently recruit Polo. Proteins recruiting Polo are indicated in shades of blue; proteins not recruiting Polo are indicated in black. Black arrows indicate how the phosphorylation of one protein can recruit Polo and so lead to the phosphorylation of the next protein in the putative cascade. See main text for details. (C) Schematic illustrates the genealogy of the centrosomes analysed for their ability to recruit GFP–Cnn from one cycle to the next. In the first division cycle, the centrosome with the OM is associated with a larger GFP–Cnn scaffold than the centrosome with the new mother centriole (NM1) (Conduit et al., 2010). When these centrosomes divide, the OM and NM1 centrosomes each generate a new centrosome containing a younger mother centriole (that is again smaller than the centrosome containing the original mother centriole) – NM2 and NM3, respectively. (D,E) Examples of OM1 and NM1 centrosomes generated at the start of the first cycle, and the NM2 and NM3 centrosomes they generated at the end of the second cycle in (D) WT-Ana1–mCherry or (E) Ana1-S34T–mCherry (magenta) embryos expressing GFP–Cnn (green). In Ana1-S34T–mCherry embryos the centrosome pairs (particularly NM1 and NM3) sometimes failed to separate properly. (F) Graph shows the mean GFP–Cnn intensity at each centrosome type in WT-Ana1–mCherry (black and grey triangles) and Ana1-S34T–mCherry (green circles) embryos. *N*=5 and 8 embryos analysed for WT-Ana1 and Ana1-S34T genotypes, respectively; three pairs of centrosomes in the first cycle were analysed per embryo, so a total of *n*=15 and 24 centrosome pairs for the WT-Ana1 and Ana1-S34T genotype, respectively (note that for the Ana1-S34T genotype, only 23 OM–NM2 pairs and 19 NM1–NM3 pairs could be analysed, due to the lack of centrosome separation at the beginning of the second cycle). To facilitate visualisation, only the *P-*values corresponding to the most informative statistical comparisons are shown, coloured by the type of centrosome being compared against others: WT NM1 in the second cycle (navy blue), S34T OM in the second cycle (magenta), and S34T NM1 in the second cycle (gold). (G) Graph shows the same data as in F, but expressed as the average sum of GFP–Cnn levels for OM and NM1 centrosomes in the first cycle (dark grey for WT-Ana1–mCherry embryos, dark green for Ana1-S34T–mCherry embryos), and the average sum of GFP–Cnn levels for OM, NM1, NM2 and NM3 centrosomes in the second cycle (light grey for WT-Ana1–mCherry embryos, light green for Ana1-S34T–mCherry embryos). Error bars represent s.d. *P*-values were calculated using an ordinary one-way ANOVA with Tukey's multiple comparison test. a.u., arbitrary units.

We wanted to better understand this puzzling heterogeneity in PCM scaffold assembly in Ana1-S34T embryos (because, unlike in our mRNA injection experiments, these embryos completely lack endogenous WT Ana1, so the centrioles cannot contain different levels of the WT and mutant protein). We reasoned that the Polo initially recruited to centrioles by the single T200 motif in Sas-4 (Novak et al., 2016) might normally be insufficient to phosphorylate Spd-2 to a high enough level to initiate the positive feedback loop that drives PCM scaffold expansion. If so, this Polo might normally phosphorylate Ana1 to create additional PBD-binding sites that can then recruit more Polo, which can then phosphorylate Spd-2 to a sufficient level to drive PCM scaffold assembly (Fig. 6A). In such a scenario, the centrioles in Ana1-S34T embryos would fail to expand a mitotic PCM scaffold because they cannot recruit sufficient Polo to initiate the feedback loop (Fig. 6B, panel i). Perhaps some of these centrioles, however, can eventually bypass the requirement for Ana1-dependent Polo recruitment if, for example, the Polo recruited by Sas-4 can eventually phosphorylate Spd-2 to a high enough level to initiate the feedback loop (Fig. 6B, panel ii). If so, then, once activated, the feedback loop might be self-sustaining – so the occasional centrosome that managed to organise significant amounts of PCM in Ana1-S34T embryos would continue to do so through repeated rounds of division. To test if this was the case, we performed a pedigree analysis of dividing centrosomes in living WT-Ana1–mCherry and Ana1-S34T–mCherry embryos co-expressing GFP–Cnn.

GFP–Cnn was initially asymmetrically distributed on centrosome pairs in both WT-Ana1–mCherry and Ana1-S34T–mCherry embryos, consistent with previous reports that OM centrioles initially associate with more GFP–Cnn than NM centrioles (Conduit et al., 2010) (Fig. 6C,D). We therefore refer to the larger centrosome as the OM, and the first smaller centrosome that it generates as the first NM (NM1). In WT-Ana1–mCherry embryos, the OM centrosomes divided again to generate a second new mother (NM2), while the original NM1 centrosome divided again to generate a new NM centrosome (NM3). Importantly, both of the new centrosome pairs (OM–NM2 and NM1–NM3) were of a similar size and exhibited a similar size asymmetry to the original OM–NM1 pair (Fig. 6D,F), indicating that both the OM and NM centrioles recruited significant amounts of GFP–Cnn prior to their division. This can be seen by comparing the sum amount of GFP–Cnn at the original centrosomes (OM+NM1) to that at the four duplicated centrosomes (OM+NM1+NM2+NM3) (Fig. 6G).

In Ana1-S34T–mCherry embryos, we selected for analysis the OM centrosomes that were associated with the most GFP–Cnn, but even these centrosomes had lower levels of GFP–Cnn than the OM centrosomes in WT-Ana1–mCherry embryos, and the NM1 centrioles were associated with even lower levels (Fig. 6E,F). Thus, even the OM centrioles that recruit the most PCM in these Ana1-S34T mutant embryos cannot do so to normal levels. Moreover, the centrosomes in these embryos recruited very little additional GFP–Cnn prior to their division (Fig. 6G). Nevertheless, the OM centrioles that associated with the highest levels of GFP–Cnn consistently retained high levels after their division, while generating NM2 centrosomes that again associated with much less GFP–Cnn (Fig. 6E,F). In contrast, the NM1 centrosomes did not grow significantly prior to division, and generated NM3 centrosomes that were associated with even less GFP–Cnn (and that, presumably as a consequence, sometimes failed to separate properly from their mothers) (Fig. 6E). We conclude that the ability of some centrioles to organise appreciable amounts of PCM in Ana1-S34T embryos is a heritable characteristic. We performed a similar pedigree analysis in living WT-Ana1–mCherry and Ana1-S34T–mCherry embryos co-expressing Spd-2–GFP and obtained very similar results (Fig. S4). These findings support our hypothesis that the ability of Ana1 to recruit Polo is required for efficient PCM assembly, and that some older centrioles can eventually bypass this requirement by activating a self-sustaining feedback loop that can support a lower level of mitotic PCM recruitment (Fig. 5B, panel ii).

### The C-terminal region of Ana1 is required to recruit Polo to centrioles

**Fig. 7.**
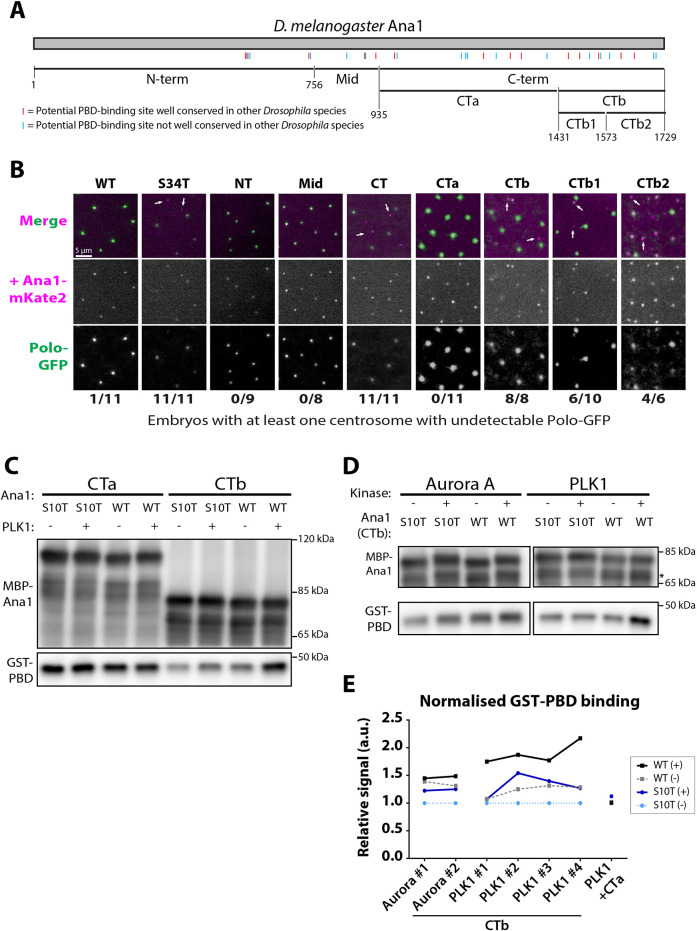
**The C-terminal region of Ana1 helps recruit Polo to centrosomes *in vivo* and can interact directly with the Polo PBD *in vitro*.** (A) Schematic representation of the protein sequence of *Drosophila melanogaster* Ana1 indicating S-S/T motifs that are either conserved (present in at least 11/12 *Drosophila* species analysed, red lines) or not conserved (blue lines). The boundaries of the Ana1 fragments we analysed are indicated, with numbers indicating amino acid positions. (B) Micrographs of embryos expressing Polo–GFP (green in the merged images) injected with constructs encoding full-length Ana1–mKate2 (WT), or Ana1–mKate2 constructs in which either the whole protein (S34T), or only the various Ana1 sub-regions – as indicated in A – have had their S-S/T motifs replaced with T-S/T (magenta in the merged images). Arrows highlight some of the centrosomes that do not recruit Polo–GFP. Embryos were scored positive if they showed loss of Polo–GFP (scored blind) from at least one centrosome; the results for each injected construct are indicated numerically as affected embryos/total injected embryos analysed. Note that the CTa, CTb, CTb1 and CTb2 injections were performed at a later date on a different microscope system, so the images look different to the others presented in this paper. (C) Western blots of an *in vitro* assay in which purified recombinant MBP fusion proteins to either WT or mutant (S10T) CTa or CTb were incubated with or without PLK1 and then assessed for their ability to bind to recombinant human GST–PBD. Only CTb exhibited specific binding (i.e. binding was enhanced by PLK1 phosphorylation, and this depended on the S-S/T motifs). (D) Same as C, except in this experiment we tested the ability of WT or mutant MBP–CTb to bind to GST–PBD after phosphorylation by either Aurora A or PLK1. Only WT CTb phosphorylated by PLK1 exhibited specific binding. Asterisk indicates a smaller band, presumably a partial degradation product of the fusion protein. (E) Graphs quantify the level of GST–PBD binding to the different MBP–CTb fusion proteins in two (Aurora A) or four (PLK1) technical repeats of these *in vitro* binding assays, and one technical repeat using CTa and PLK1 (a.u., arbitrary units). Although these assays are somewhat variable, the WT CTb fragment consistently exhibits elevated levels of binding to GST–PBD when it is phosphorylated by PLK1.

Ana1 is asymmetrically organised at centrioles, with its N terminus located towards the central core and its C terminus extending outwards towards the centriole periphery (Fu et al., 2016) where the PCM is recruited (Fu and Glover, 2012; Lawo et al., 2012; Mennella et al., 2012; Sonnen et al., 2012). To test which regions of Ana1 might be most important for Polo recruitment, we generated mutant forms that contained S-to-T substitutions in only the N-terminal (amino acids 1–756), mid (amino acids 756–935) or C-terminal (amino acids 935–1729) regions (Fig. 7A) and tested their ability to recruit Polo using the mRNA injection assay. Only the C-terminally mutated protein (containing 20 S-to-T substitutions) perturbed Polo–GFP recruitment (Fig. 7B). We further subdivided the C-terminal region into CTa and CTb regions (containing ten S-S/T motifs each), and found that only C-terminal CTb mutations strongly perturbed Polo–GFP recruitment (Fig. 7A,B). Finally, we subdivided CTb into CTb1 and CTb2 (each containing five S-S/T motifs) and found that both mutant proteins partially perturbed Polo–GFP recruitment, but not as strongly as CTb. Taken together, these findings suggest that multiple S-S/T motifs in the C-terminal region of Ana1 contribute to recruiting Polo to centrioles *in vivo* (Fig. 7B).

To test whether these C-terminal S-S/T motifs could interact with the PBD *in vitro*, we expressed and purified MBP-tagged fusions containing WT or S-to-T substitutions of the CTa fragment (which does not help recruit Polo *in vivo*) and the CTb fragment (which does help to recruit Polo *in vivo*). We pre-treated the MBP fusions with either buffer or recombinant human PLK1 and then tested whether they could bind to recombinant GST–PBD. CTa could bind to GST–PBD, but this binding was non-specific – it was not dependent on the presence of the S-S/T motifs, nor was it enhanced by PLK1 phosphorylation (note that, in our hands, the vast majority of the many MBP fusion proteins we have tested can bind to GST–PBD non-specifically in this assay to varying degrees) (Fig. 7C,E). In contrast, CTb exhibited enhanced binding to GST–PBD when it had been phosphorylated by PLK1 *in vitro*, and this enhancement depended on the presence of the S-S/T motifs. As a further control, we also tested whether MBP–CTb could be induced to bind to GST–PBD after it had been phosphorylated by Aurora A *in vitro*. Although Aurora A seemed to phosphorylate both the WT and mutant form of MBP–CTb (as evidenced by the slight band shift on the gel), this phosphorylation did not result in enhanced binding to GST–PBD (Fig. 7D,E).

We remain cautious in interpreting these results, as these *in vitro* binding assays are somewhat variable (Fig. 7E) and may not accurately reflect the situation *in vivo*. Nevertheless, they indicate that the C-terminal region of Ana1 contains S-S/T motifs that, when phosphorylated by PLK1, can directly bind to the PBD *in vitro*, and so could plausibly play a part in recruiting Polo to centrioles when they are phosphorylated by Polo *in vivo*. Moreover, the ability of PLK1 to potentially ‘self-prime’ its own recruitment (Kang et al., 2006; Neef et al., 2003, 2007; Decker et al., 2011; Alvarez-Rodrigo et al., 2019) is consistent with our hypothesis that the Polo initially recruited to centrioles by Sas-4-T200 might phosphorylate Ana1 to allow it to recruit additional Polo to the centriole.

### The ability of Ana1 to promote centriole growth appears to require its ability to recruit Polo

It has previously been shown that the Ana1 and Cep295 proteins promote centriole growth (Chang et al., 2016; Saurya et al., 2016), potentially by acting downstream of Ana3 and Rotatin, respectively, to stimulate the growth of the centriole MTs as they extend distally past the central cartwheel structure (Chen et al., 2017). This ‘second phase’ of centriole growth occurs largely in G2, and in human cells it requires PLK1 (Kong et al., 2020). The centrioles in most fly cells are very small and extend only slightly during G2, but our EM analysis revealed that overexpressing WT-Ana1–GFP led to a small, but significant, increase in the length of these centrioles, whereas overexpressing Ana1-S34T–GFP did not (Fig. 2D,E). Thus, the ability of Ana1 to recruit Polo might be important for promoting this second phase of centriole growth during G2.

**Fig. 8.**
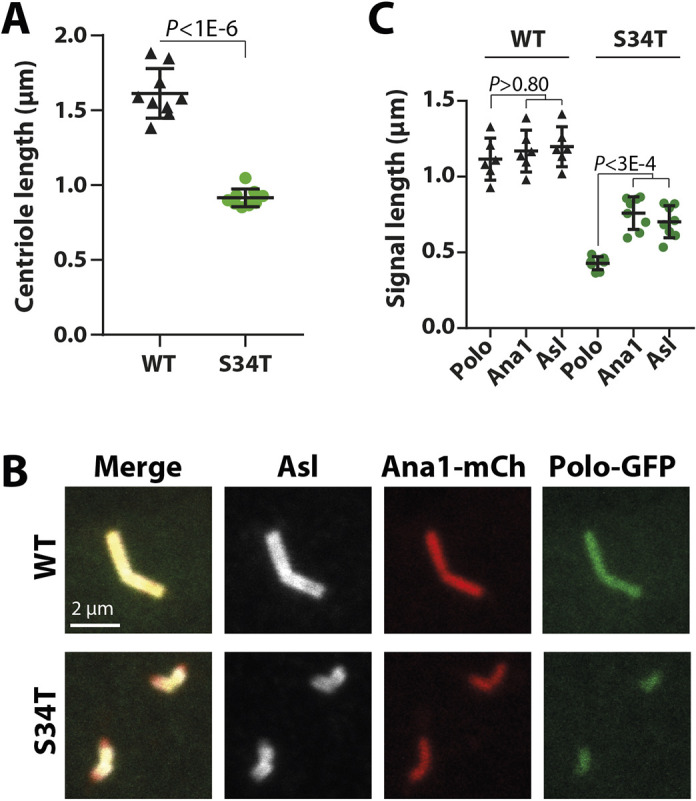
**Ana1 helps to recruit Polo to the centriole distal end to promote centriole elongation.** (A) Graph quantifies centriole length in *ana1^−/−^* mutant testes expressing either WT-Ana1–GFP or Ana1-S34T–GFP. Each data point represents an individual testis and shows the average centriole length calculated from >10 centrioles. Error bars indicate the s.d. (B) Micrographs show typical centriole pairs in fixed *ana1^−/−^* mutant spermatocytes expressing Polo–GFP (green) and either WT-Ana1–mCherry (top panels) or Ana1-S34T–mCherry (bottom panels) (Ana1–mCh; red) stained to reveal the distribution of the centriole protein Asl (white). The centriole pairs in spermatocytes are engaged and arranged in a characteristic V-shape, and they grow to a much longer length than the centrioles in most other *Drosophila* cell types during an extended G2 period. In the Ana1-S34T–mCherry spermatocytes, the centriole pairs are duplicated and arranged in the typical V-shape, but they are much shorter than normal. In the WT centrioles the Asl, Ana1 and Polo extend along the entire length of the extended centrioles, whereas in the S34T centrioles Polo does not extend outwards as far as the centriole distal end, suggesting that Ana1 is normally required to recruit Polo to the centriole distal end. (C) Graph quantifies the length that the Asl, Ana1 and Polo signals spread outwards along the centrioles in the experiment described in B. Each data point represents an individual testis and shows the average spread of each protein calculated from >10 centrioles. Error bars indicate the s.d. Note how in WT centrioles Asl, Ana1 and Polo spread along the entire length of the centriole to the same extent, while in the S34T centrioles Polo specifically does not extend to the distal end. *P*-values in A and C were calculated using an unpaired two-tailed *t*-test with Welch correction and the ordinary one-way ANOVA with Tukey's multiple comparison test, respectively.

The centrioles in spermatocytes (which go on to form the sperm flagella) exhibit a much more pronounced phase of growth during G2 (Tates, 1971), and this requires Ana1 (Saurya et al., 2016). In *ana1^−/−^* mutant spermatocytes expressing Ana1-S34T–GFP the centrioles were much shorter than those in spermatocytes rescued with WT-Ana1GFP (Fig. 8A). These shortened centrioles appeared to duplicate, disengage and separate normally, but the basal body of the spermatids (formed after the spermatocytes had proceeded through meiosis) were significantly shorter than normal, and the males exhibited reduced fertility (Fig. S5). In *ana1^−/−^* mutant spermatocytes rescued with WT-Ana1–mCherry, Asl and Polo–GFP were recruited along the entire length of the centrioles (Fig. 8B,C). Conversely, in the shortened centrioles of *ana1^−/−^* mutant spermatocytes expressing Ana1-S34T–mCherry, Polo–GFP no longer extended to the distal ends (Fig. 8C). Taken together, these data suggest that Ana1 is required to recruit Polo to the distal end of centrioles to promote centriole elongation during G2.

## DISCUSSION

Polo has many important functions at centrioles and centrosomes, and we previously have shown that it is initially recruited to newborn centrioles in flies when Cdk1 phosphorylates the Sas-4 T200 S-T motif during mitosis. This initial recruitment of Polo is important to allow the newborn centrioles to subsequently mature into mothers that can recruit Asl and so duplicate and recruit mitotic PCM (Novak et al., 2016). Here, we show that the centriole protein Ana1 also plays an important part in recruiting Polo to mother centrioles. Our data suggests that Ana1 can recruit Polo directly and that Polo itself can phosphorylate Ana1 at several S-S/T motifs to ‘self-prime’ its own recruitment. We cannot exclude, however, that other protein kinases may prime these S-S/T motifs, or that Ana1 could recruit Polo to centrioles indirectly in ways that are disrupted when the S-S/T motifs are mutated to T-S/T. Regardless of mechanism, the Ana1-dependent centriolar pool of Polo appears to be required to drive efficient mitotic PCM expansion and centriole elongation in G2.

Although Ana1 helps recruit and/or maintain Asl at centrioles – and so is essential for both mitotic PCM recruitment and centriole duplication (Chang et al., 2016; Fu et al., 2016; Izquierdo et al., 2014; Knorz et al., 2010; Saurya et al., 2016; Tsuchiya et al., 2016) – this function of Ana1 does not appear to require the ability to recruit Polo. Thus, Ana1-S34T centrioles recruit and maintain normal levels of Asl (and of Cep135, as well as slightly increased levels of Sas-4) and can duplicate normally. This is in contrast to the situation with Sas-4, where T200 phosphorylation is required for proper Asl recruitment and so for both centriole duplication and mitotic PCM assembly (Novak et al., 2016). Presumably, the Polo recruited by Sas-4 is either sufficient for Asl recruitment, or it phosphorylates centriole substrates other than Ana1 to promote Asl recruitment. Interestingly, PLK1 is also essential for efficient centriole disengagement (Kong et al., 2014; Loncarek et al., 2010; Tsou et al., 2009; Shukla et al., 2015), but neither the Ana1-S34T nor Sas-4-T200 mutations appear to perturb this process, indicating that a separate pathway must recruit Polo to centrioles to drive centriole disengagement. Centrosome separation in G2 is also normally dependent on PLK1 (Bertran et al., 2011; Smith et al., 2011; Mardin et al., 2011), and we often observed centrosomes/duplicated centriole pairs that failed to separate properly in embryos expressing Ana1-S34T (Fig. 6E; Fig. S2). As these centriole pairs almost always organised very little PCM, however, we suspect that this defect may be an indirect consequence of the failure to properly recruit PCM, rather than a direct consequence of the inability of Ana1 to recruit Polo.

These new findings further support our hypothesis that centrioles activate a Spd-2–Polo–Cnn positive feedback loop that drives the expansion of the mitotic PCM around the mother centriole. A key feature of this model is that Spd-2 can only be phosphorylated to initiate scaffold assembly at the surface of the mother centriole, and the phosphorylated Spd-2 then fluxes outwards away from the centriole: the Spd-2–Polo–Cnn scaffold itself cannot phosphorylate and/or recruit new Spd-2 into the scaffold (Conduit et al., 2014b). This is important, as it can explain why the mother centriole is required to drive efficient mitotic PCM assembly (Bobinnec et al., 1998; Basto et al., 2006), why the size of the centriole influences the size of the mitotic PCM (Kirkham et al., 2003) and why centrioles are constantly required to drive the growth of the mitotic PCM (Cabral et al., 2019). All of these findings can be explained if the mother centriole is the only source of the phosphorylated Spd-2 scaffold. Our observation that the pool of Polo recruited by Ana1 – which, unlike Spd-2, is not a PCM component and is restricted to the centriole – is required for the efficient expansion of the PCM demonstrates that the PCM-associated pool of Polo (recruited by Spd-2) is not sufficient to drive efficient PCM expansion on its own. It is important to stress, however, that so far an outward flux of Spd-2 from the centriole has only been observed in fly embryos and cells (Conduit et al., 2014b; Conduit and Raff, 2015) and has not been detected for SPD-2 in *C. elegans* embryos (Cabral et al., 2019). Clearly it will be important to establish whether such a Spd-2 or Cep192 flux exists in other species.

The ability of Ana1 to recruit Polo also appears to be required for centriole elongation during G2. In human cells, PLK1 is required for this process (Kong et al., 2020), although a previous study did not report any change in centriole length after long-term Polo-inhibition in fly spermatocytes (Riparbelli et al., 2014). Clearly more work is required to establish whether Polo recruitment by Ana1 has a role in G2 centriole elongation in flies, as our work suggests, and, if so, what Polo's relevant substrates are at the centriole distal end.

Finally, we note that both the Ana1/Cep295 and Spd-2/Cep192 protein families have a relatively high density of potential PBD-binding sites (S-S/T motifs) when compared to several other centriole and centrosome proteins (Fig. S6). This suggests that these proteins might have evolved to function as scaffolds that amplify Polo levels at specific locations within the cell during mitosis. It will be interesting to examine whether other proteins with a high density of potential PBD-binding domains serve a similar function at other locations within the mitotic cell. Our strategy of mutating all S-S/T motifs to T-S/T in candidate proteins may be a good way of testing this possibility as, for both Ana1 and Spd-2 at least, the S-to-T substitutions seem to specifically impair Polo-recruitment without more generally perturbing the function of the proteins or centriole/centrosome structure.

## MATERIALS AND METHODS

### Fly husbandry, stocks and handling

Flies were kept at 25°C or 18°C on *Drosophila* culture medium (0.77% agar, 6.9% maize, 0.8% soya, 1.4% yeast, 6.9% malt, 1.9% molasses, 0.5% propionic acid, 0.03% ortho-phosphoric acid and 0.3% nipagin). The following fly lines have been previously described: Polo–GFP protein trap (Buszczak et al., 2007), UAS-mCD8-GFP (Lee and Luo, 1999), Ubq-Sas-4-GFP (Novak et al., 2014), eAsl-mCherry (Conduit et al., 2015a), Ubq-GFP-Cep135 (Roque et al., 2012), Ubq-GFP-Cnn (Conduit et al., 2010) and Ubq-Spd-2-GFP (Dix and Raff, 2007). Embryos without endogenous Ana1 (i.e. from *ana1^−/−^* mutant females) were derived from *ana1^mecB^/Df(3R)Exel7357* hemizygous mutant mothers (Blachon et al., 2009). The mCherry and GFP Ubq-Ana1 WT or S34T transgenic lines were generated by the Fly Facility in the Department of Genetics, Cambridge (UK) via random P-element insertion of the construct of choice (containing a *w^+^* gene for selection) in to a *w118* background. Stocks were kept in 8 cm×2.5 cm plastic vials or 0.25-pint plastic bottles. *Drosophila melanogaster* Oregon-R flies were used as a WT stock for EM and western blotting.

Embryos were collected on cranberry–raspberry juice plates (25% cranberry–raspberry juice, 2% sucrose and 1.8% agar) supplemented with fresh yeast. Standard fly handling techniques were employed (Roberts, 1998). *In vivo* studies were performed using 1.5–2-h-old syncytial blastoderm stage embryos. After 0–1 h collections at 25°C, embryos were aged at 25°C for 30–60 min. When injecting mRNA, embryos were collected for 20 min, injected and imaged after 120–150 min at 21°C (but always within the syncytial blastoderm stage of development). Prior to injection or imaging, embryos were dechorionated on double-sided tape and mounted on a strip of glue onto a 35 mm glass bottom Petri dish with a 14 mm micro-well (MatTek). After desiccation for 1 min (non-injection experiments) or 3 min (pre-mRNA injection) at 25°C, embryos were covered in Voltalef oil (ARKEMA). Live imaging was performed using either the spinning disk confocal or the 3D-SIM systems described below.

### Generation of Polo-binding site mutants

Potential Polo-binding sites in the amino acid sequence of the candidate centrosomal proteins were identified by searching for the consensus Polo-binding motif S-S/T. Site conservation was assessed using FlyBase BLAST (selecting the genus *Drosophila*) and Jalview (Waterhouse et al., 2009) for protein alignment. The mutant constructs were designed *in silico* and synthesised externally by GENEWIZ Co. Ltd. (Suzhou, China); the WT cDNAs were obtained from the *Drosophila* Genomics Resource Centre, USA. All cDNAs were cloned into a pDONR-Zeo vector and then introduced via Gateway cloning (Thermo Fisher Scientific; 11789100 and 11791100) in pRNA-mKate2CT (Novak et al., 2016) or Ubq-GFPCT and Ubq-mCherryCT (Basto et al., 2008) destination vectors, as indicated. NEBuilder HiFi assembly (NEB; E2621S) was used to produce pRNA-mKate2 plasmids encoding Ana1 ‘partial mutants’ and to introduce fragments encoding WT or mutant Ana1 amino acids 1431–1729 into a pETM44 (EMBL) vector encoding an N-terminal His6–MBP tag.

### RNA synthesis and microinjection

The mRNA injection assay has been described previously (Novak et al., 2014). *In vitro* RNA synthesis was performed using a T3 mMESSAGE mMACHINE kit (Thermo Fisher; AM1348) and RNA was purified using an RNeasy MinElute kit (Qiagen; 74106) according to the manufacturer's instructions. All RNA constructs were stored at −80°C and injected at a concentration of 2 mg/ml.

### Behavioural assays

#### Hatching experiments

To examine the quality of the embryonic development for various fly strains generated in this study, 1–5 h collected embryos were aged for 24 h, and the percentage of the embryos that had hatched out of their chorion was calculated. At least two technical repeats per transgenic fly line (GFP- and mCherry-tagged) were performed.

#### Negative gravitaxis experiments

A standard negative gravitaxis assay was used to assess the climbing reflexes of *ana1^−/−^* mutant flies (Ma and Jarman, 2011; Pratt et al., 2016). Fifteen 1–3-d-old adult male flies were sharply tapped to the bottom of a 10 ml cylinder, and the maximum distance climbed by individual flies within the first 5 s after tapping was measured. The distances were calculated using Fiji (ImageJ; https://fiji.sc/). Measurements were repeated four times (technical repeats) for each transgenic fly line (GFP- and mCherry-tagged).

#### Fertility assays

Individual 3–5-d-old *ana1^−/−^* rescued males were crossed to two Oregon-R virgin females each. The crosses were knocked into fresh vials 2 d and 4 d after setting the original cross. All vials were kept at 25°C. The number of individuals born from each vial was counted 17 d after setting the original cross.

### Transmission electron microscopy

Wing-discs from third-instar larvae were prepared as described previously (Stevens et al., 2010) with slight modifications. Briefly, WT wing discs and the *ana1*^−/−^ rescued fly wing discs and brains were dissected in phosphate-buffered saline (PBS) and fixed in 2.5% glutaraldehyde and 4% paraformaldehyde in 0.1 M in PIPES buffer (pH 7.2) for 1 h (up to 2 h) at room temperature then left overnight in the fridge at 4°C. Samples were then washed twice in 0.1 M PIPES, followed by one wash in 50 mM glycine in 0.1 M PIPES to quench free aldehydes, and then another wash in 0.1 M PIPES. Samples were then post-fixed in 1% OsO_4_ for 2 h at room temperature, followed by extensive washing in distilled water. Samples were then stained with 0.5% uranyl acetate overnight at 4°C, washed in distilled water, dehydrated in an ethanol series and embedded in Agar100 (Agar Scientific). Blocks were polymerised at 50°C for 24–42 h. Semi-thin serial sections (100 nm) were obtained in a Leica EM UC7 ultramicrotome (Leica Microsystems, Austria) and stained using lead citrate. Images of centrioles were taken on a TECNAI T12 transmission microscope (FEI, The Netherlands) at 13,000× magnification, to measure centriole length from wing discs. The length of the MT doublets within the electron-dense area was measured using the line tool in Fiji (ImageJ, version 2.0.0-rc-69/1.52i).

### Western blot analysis

Western blotting to estimate embryonic protein levels and the results from the *in vitro* interaction assays was performed as described previously (Novak et al., 2014). The following primary antibodies were used: rabbit anti-Ana1 (1:500; animal #SK4818; Stevens et al., 2009), mouse anti-Actin (1:500; Sigma; A3853) and mouse anti-GST (1:500; Thermo Fisher Scientific; MA4-004). For visualisation, we used the SuperSignal West Femto kit (Thermo Fisher Scientific; 34095) and the following HRP-conjugated secondary antibodies: swine anti-rabbit immunoglobulins (1:3000 for embryo levels, 1:10,000 for *in vitro* assays; Dako; P0399) and sheep ECL anti-mouse IgG (1:3000; GE Healthcare; NA931V).

### Immunofluorescence

Embryos were collected for 1 h, aged for 1 h, and processed as described previously (Gartenmann et al., 2020). Testes from adult male flies expressing either WT or S34T Ana1–GFP constructs in an *ana1^−/−^* background were dissected, fixed and stained, as described previously (Roque et al., 2012). Samples were mounted onto microscopy slides with high-precision glass coverslips (CellPath). The following antibodies were used: mouse anti-α-tubulin (1:1000; Sigma; DM1a), guinea pig anti-Cnn antibody (1:1000; animal #SK3516; Lucas and Raff, 2007), rabbit anti-Cnn pSer567 antibody (1:500; animal #30129; Feng et al., 2017), guinea pig anti-Asl (1:500; animal # SKC124; Roque et al., 2012), Alexa Fluor 647 nm-conjugated anti-mouse IgG (1:500; Thermo Fisher Scientific; A21236), GFP-Booster Atto488-conjugated anti-GFP (1:500; Chromotek; gba488), CF405S-labelled anti-guinea pig IgG (1:500; Biotium; 20356), Alexa Fluor 488 nm-conjugated anti-rabbit IgG (1:500; Thermo Fisher Scientific; A21206) and Alexa Fluor 568 nm-conjugated anti-guinea pig IgG (1:500; Thermo Fisher Scientific; A11075). For quantification of mitotic defects and centriole length in testes, we used Vectashield medium with DAPI (Vector Laboratories; H-1200), whereas for the Cnn staining, we used Vectashield medium without DAPI (Vector Laboratories; H-1000).

### Light microscopy and image analysis

#### Spinning disk confocal microscopy

Embryos, third-instar larval brains and adult antennae were imaged at 21°C on a Perkin Elmer ERS spinning disk (Volocity software version 6.3; PerkinElmer Inc.) mounted on a Zeiss Axiovert 200M microscope using a 63×/1.4-NA oil immersion objective and an Orca ER CCD camera (Hamamatsu Photonics). 488- and 561-nm lasers were used to excite GFP and mKate2/mCherry, respectively. Confocal sections of 13 slices with 0.5-μm-thick intervals were collected every 30 s. Focus was occasionally manually readjusted between intervals. For Fig. 4A,C and Fig. 7B (CTa-CTb2), embryos were imaged using an Andor Dragonfly 505 spinning disk mounted on a Leica DMi8 microscope with a HC PL APO 63×/1.40 oil immersion objective and an Andor iXon Ultra 888 EMCCD camera.

For the quantification of centrosomes in neuroblasts, *ana1^−/−^* mutant larvae expressing Spd-2–GFP and Ana1–mCherry (either WT or S34T) were blinded and randomised prior to dissection, imaging and scoring. During live imaging, mitotic centrosomes in neuroblasts were identified by the presence of at least one dot of colocalised Spd-2–GFP and Ana1–mCherry and scored. A total of 2–4 neuroblasts were scored per brain (total number of neuroblasts and brains scored are indicated in the corresponding figure).

For quantification of centrosomal protein levels in the injection assay (Fig. 1E,F) and *ana1^−/−^* embryos expressing Ana1 transgenes, we measured the mean intensity within a square of fixed size centred manually on each individual centrosome, and the mean intensity of the background near each centrosome. We then calculated the average centrosome intensity and subtracted the average background intensity per embryo. The number of embryos analysed is indicated in the corresponding figure legends. For embryos expressing only Ana1–GFP (Fig. S1C), we used the maximum intensity projection of the *z*-stack, analysed ten pairs of centrosomes per embryo, and classified the data into two subsets: OM (data from the brightest centrosome from each pair) and NM (data from the other centrosomes). However, to analyse embryos co-expressing two different fluorescent proteins, the protocol was adapted as required (see below).

For embryos in the candidate screen assay and embryos co-expressing an Ana1 transgene and GFP–Cep135, Asl–mCherry, Sas-4–GFP or Polo–GFP; we could not use the maximum intensity projection of the *z*-stack, and instead we selected the *z*-slice where the most centrosomes were in focus. If possible, the images were blinded prior to quantification and the data was classified into OM and NM subsets based on Asl or Ana1 levels (as indicated). For embryos co-expressing Polo–GFP and Ana1–mCherry, we analysed all centrosomes containing Ana1-S34T–mCherry (i.e. a total of 23 Ana1-S34T–mCherry foci from six different embryos) and quantified the mean intensity of the corresponding area in the 488 nm channel. As this was on average four centrosomes/embryo, from every WT-Ana1–mCherry control embryo we analysed two random pairs of centrosomes. As it was not possible to distinguish embryo age or cell cycle stage in the S34T-rescued embryos, we analysed a panel of control embryos as diverse as possible: embryos at nuclear cycle 10 to 14, and at early S phase, late S phase and metaphase/anaphase. The data is shown as an average of all analysed centrosomes together (Fig. 3C,D) or following classification of the control centrosomes by developmental or cell cycle stage (Fig. S3).

For embryos co-expressing Ana1–mCherry and GFP–Cnn or Spd-2–GFP, we selected random pairs of separating centrosomes at the beginning of the first nuclear division imaged based solely on the Ana1–mCherry images (to avoid selection bias) and manually tracked the pairs until the following S phase. If the tracking was successful, both pairs of centrosomes were visible and at least one of the two pairs visibly separated, that family of centrosomes was included in the analysis. Centrosomes were classified by types according to the pedigree schematic in Fig. 6C, with the older mother centrosomes corresponding to those with the highest GFP–Cnn or Spd-2–GFP intensity. Three families of centrosomes were analysed per embryo, and the total number of centrosomes and embryos analysed is indicated in the corresponding figure. The results were plotted as an average per type of centrosome (OM, NM1, NM2 or NM3; in the first or second cycle imaged) (Fig. 6F; Fig. S4C); or as an average cumulative intensity (Fig. 6G; Fig. S4D). The cumulative intensity is the sum of the intensity values for OM and NM1 centrosomes in the first cycle analysed; or the sum of OM, NM1, NM2 and NM3 values in the second cycle.

In addition to the quantification described above, the effect of injecting different mutant versions of Ana1–mKate2 into Polo-GFP embryos (Fig. 1E,F and Fig. 7B) was scored as follows: a two-colour *z*-slice with the most centrosomes in focus was selected per embryo, the images from all the different conditions tested were blinded and randomised, and one independent scorer was asked to determine for each image whether all the centrosomes visible in the red channel (i.e. those that had incorporated WT or mutant Ana1–mKate2) were also visible in the green channel (i.e. had visible amount of Polo–GFP).

#### Quantification of mitotic defects

Fixed samples were imaged using an inverted Zeiss 880 microscope fitted with an Airyscan detector (Zeiss International, Micron Oxford). The system was equipped with Plan-Apochromat 63×/1.4-NA oil lens. The laser excitation lines used were 405 nm diode, 488 nm argon and 633 nm diode laser. Stacks of 25 slices with 0.14 μm intervals were collected with pixel size (*x,y*) of 0.035 μm, using a piezo-driven *z*-positioner stage. Images were Airy-processed in 3D with a strength value of ‘auto’ (∼6). The software used to acquire images and process the images taken in super-resolution Airyscan mode was ZEN (black edition; Zeiss International). Maximum intensity projections of the images were used to count the number of centrosomes per visible pole, and the number of poles associated with each visible centrosome. One image analysed per embryo, 6–11 embryos analysed (from a panel of embryos at different points of cell cycle and syncytial stages, as it was difficult to accurately identify cell cycle and syncytial stages in Ana1-S34T–GFP embryos).

#### 3D-SIM

3D-SIM microscopy was performed and analysed as described previously (Conduit et al., 2014b) on an OMX V3 Blaze microscope (GE Healthcare, Micron Oxford, 29065721) with a 60×/1.42-NA oil UPlanSApo objective (Olympus); 405, 488 and 593 nm diode lasers; and Edge 5.5 sCMOS cameras (PCO). The raw acquisition was reconstructed using softWoRx 6.1 (GE Healthcare) with a Wiener filter setting of 0.006 and channel-specific optical transfer function. Living embryos were imaged at 21°C, acquiring stacks of 6 *z*-slices (0.125 μm intervals). Stacks of 13 *z*-slices (0.125 μm intervals) were acquired from fixed samples (phospho-Cnn staining). The images shown are maximum intensity projections. The images from the different colour channels were registered with alignment parameters obtained from calibration measurements using 1 μm to 0.2 μm TetraSpeck Microspheres (Thermo Fisher Scientific) using Chromagnon alignment software (Matsuda et al., 2018). The SIMcheck plug-in in ImageJ (NIH, Bethesda, MD) was used to assess the quality of the SIM reconstructions (Ball et al., 2015).

For the qualitative analysis of Spd-2 scaffold formation, centrosome images were selected based on quality of the Spd-2–GFP reconstruction, as assessed by the SIMcheck plug-in, and the presence of a visible, well-formed ring corresponding to the presence of Spd-2 at the mother centriole wall. Each individual centrosome image was saved as a separate file, and these were blinded and randomised post acquisition. The entire dataset (21 individual centrosomes per condition, two conditions) was scored independently by three different researchers not involved in any aspect of the data acquisition, and an average score was calculated.

#### Testes analysis

Fixed and stained testes slides were imaged on a confocal microscope system (FluoView FV1000; Olympus) using a 100×1.4NA oil objective and FluoView software (Olympus). Centriole length was measured using FIJI or ImageJ.

### Recombinant protein expression, purification and *in vitro* interaction assay

Proteins were expressed in *Escherichia coli* B21 strains in LB, and purified using a pre-poured amylose column containing 4 ml amylose resin (New England Biolabs; E8021L) followed by size exclusion chromatography (protein buffer: 20 mM Tris pH 8.0, 150 mM NaCl and 0.5 mM TCEP) using an AKTA pure chromatography system with a Superdex 200 10/300 GL column attached (GE Healthcare; GE17-5175-01). The *in vitro* interaction experiments using recombinant Ana1 fragments and commercial GST–PBD (Sigma; SRP0360) were carried out as described previously (Alvarez-Rodrigo et al., 2019) with the following modifications due to differences in specific kinase activity between PLK1 and Aurora A: pre-incubation at 30°C with 8.8 ng/μl of commercial PLK1 kinase (ProQinase; 0183-0000-1) or equivalent volume of PLK1 storage buffer (following manufacturer's instructions) for 90 min; or pre-incubation at 30°C with 4 ng/μl of commercial Aurora A kinase (ProQinase; 0166-0000-1) or equivalent volume of Aurora A storage buffer (following manufacturer's instructions) for 30 min.

### Statistical analysis

Prism 7 (GraphPad Software) was used for all statistical analyses. The details for quantification, statistical tests, sample numbers, the measures for dispersion and exact *P-*values are described in the main text, Materials and Methods, or corresponding figure legends. The sample size depends on embryo healthiness and how many embryos were laid per female in any particular experimental session – no explicit power analysis was used. To determine whether the data values came from a Gaussian distribution, D'Agostino–Pearson omnibus normality test was applied. To assess if the differences between means were statistically significant, we used the unpaired two-tailed *t*-test with Welch correction (when comparing two groups) or one-way ANOVA with Tukey's multiple comparison test (when comparing more than two groups).

## Supplementary Material

Supplementary information
