# Supplementary material for: Ana1 helps recruit Polo to centrioles to promote mitotic PCM assembly and centriole elongation
Source: J Cell Sci. 2021 Jul 22;134(14):jcs258987. doi: 10.1242/jcs.258987 (PMC8325959; doi:10.1242/jcs.258987)
Supplement: Supplementary information [file joces-134-258987-s1.pdf]

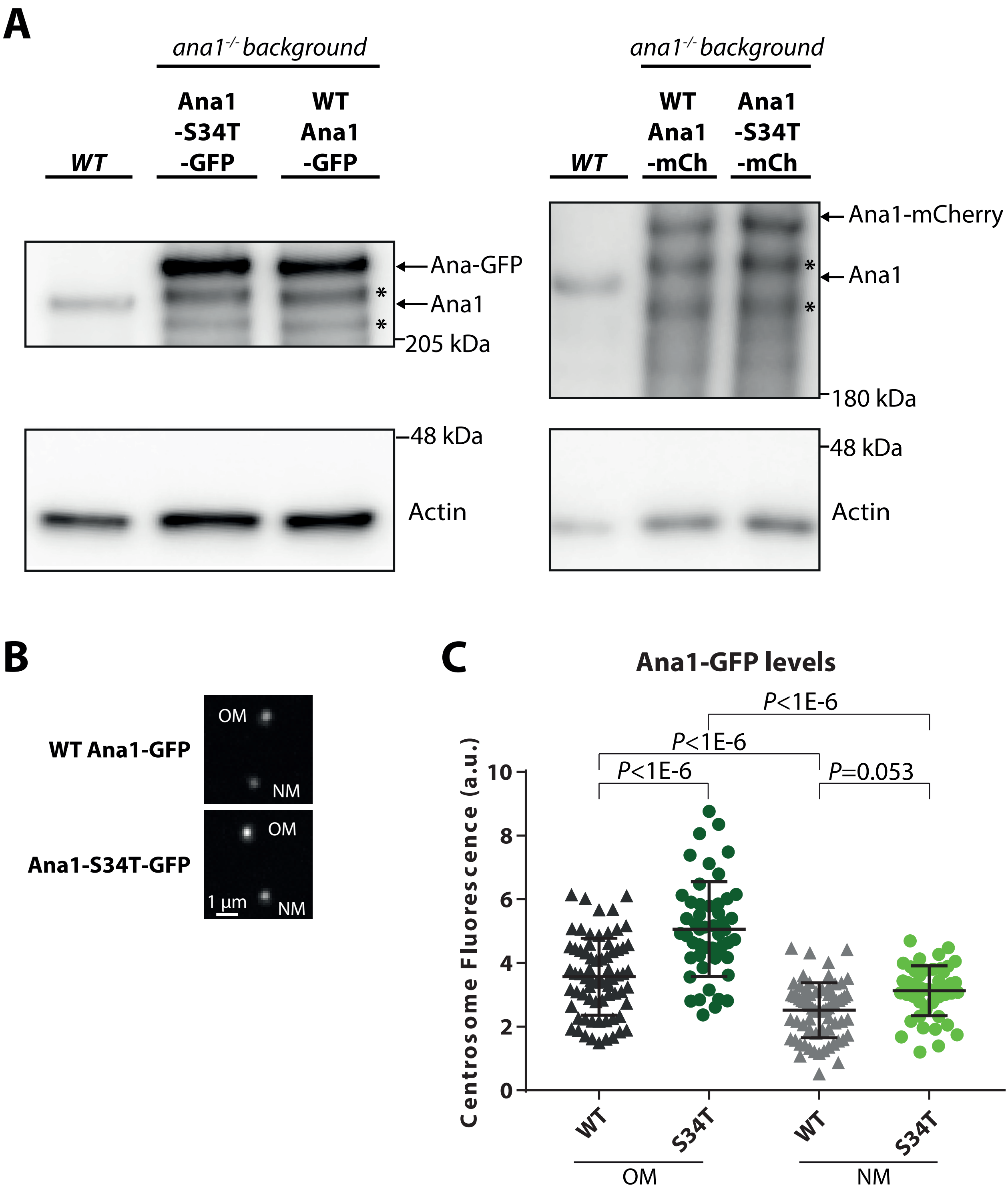

Journal of Cell Science • Supplementary information

Figure S1. Ana1-S34T-fusion proteins are expressed and localise to centrioles in a similar manner to WT Ana1-fusion proteins

(A) Western blots show total Ana1 levels in WT embryos compared to *ana1*$^{-/-}$ embryos expressing either Ana1-S34T- or WT-Ana1-fusions to either GFP or mCherry driven from the *ubiquitin* promoter. Anti-Ana1 antibodies recognise a ladder of proteins (*asterisks*) in the samples expressing the -GFP or -mCherry fusions that are likely to be degradation products. Actin is shown as a loading control. Each blot is representative of two technical repeats. **(B,C)** Micrographs show (B) and graphs quantify (C) the levels of WT-Ana1-FP and Ana1-S34T-GFP in *ana1*$^{-/-}$ embryos at the end of S-phase. 10 pairs of centrosomes were analysed per embryo (n = 70 and 50, respectively). OM and NM centrioles are quantified separately as it has previously been shown that Ana1-fusion proteins are brighter on OM centrioles (Saurya et al., 2016). Error bars represent SD.

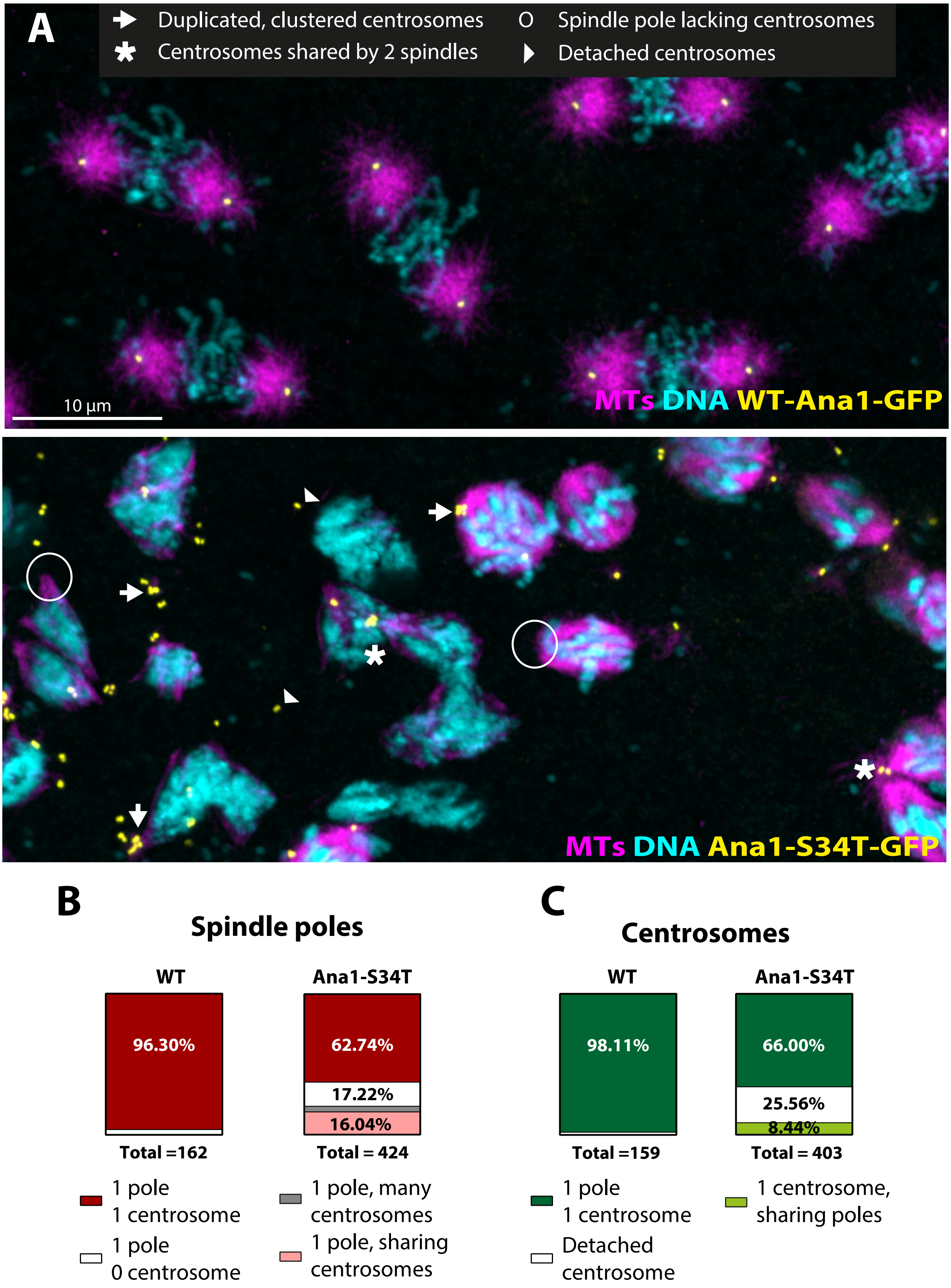

Journal of Cell Science • Supplementary information

Figure S2. Ana1-S34T-GFP embryos accumulate severe mitotic abnormalities.

(A) Representative micrographs of fixed *ana1*$^{-/-}$ embryos expressing WT Ana1-GFP (*top*) or Ana1-S34T-GFP (*bottom*) (*yellow*) were stained using an anti-α-tubulin antibody (to visualise MTs, *magenta*) and DAPI (to visualise nuclei, *cyan*). Mitotic abnormalities were rare in WT Ana1-GFP embryos but were frequent in Ana1-S34T-GFP embryos. Common defects observed are indicated in the figure legend and highlighted on the micrograph (only a few examples of each defect are marked to facilitate visualisation). **(B,C)** Quantification of some of the spindle pole (B) and centrosome (C) defects observed in eight WT and ten S34T-rescued embryos undergoing mitosis (one technical repeat). The total number of spindle poles and centrosomes scored per genotype is indicated.

Journal of Cell Science • Supplementary information

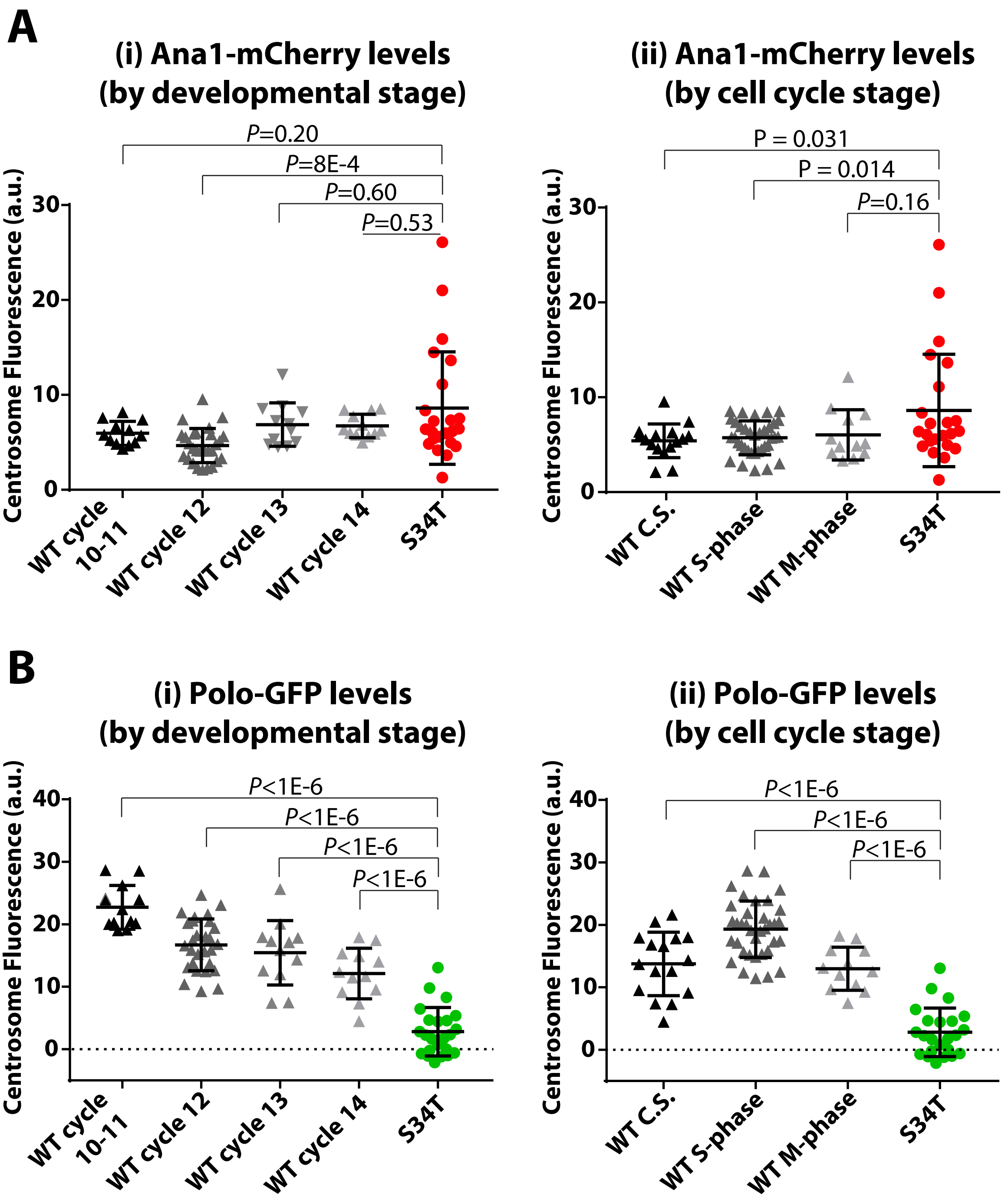

Journal of Cell Science • Supplementary information

Figure S3. Polo-GFP levels are extremely low in Ana1-S34T-mCherry embryos compared to WT-Ana1 embryos at any point of the syncytial blastoderm embryonic stage or of the cell cycle.

Graphs show the mean centrosomal Ana1-mCherry **(A)** and Polo-GFP **(B)** intensities in WT-Ana1-mCherry embryos at different nuclear cycles (*left* graphs) or at different stages of the nuclear cycle (*right* graphs), and compare this to levels in Ana1-S34T-mCherry embryos expressing Polo-GFP. Although it is difficult to accurately stage these latter embryos (as they are very sick), these graphs illustrate that the mutant Ana1-S34T-mCherry is recruited to centrosomes at similar levels to WT-Ana1-mCherry no matter what the nuclear cycle or cell cycle stage of the WT embryos, while the levels of Polo-GFP at the mutant centrosomes are significantly lower than in the WT irrespective of the nuclear cycle or cell cycle stage. For Ana1-S34T-mCherry centrosomes, n = 23 (from six different embryos); For WT-Ana1-mCherry n = 12, 28, 12, 12 (for cycles 10-11, 12, 13 and 14, respectively, from 10 embryos) and n = 16, 36, 12 (for early S-phase, mid-S-phase and mitosis, respectively, from 10 embryos). Error bars indicate SD.

Journal of Cell Science • Supplementary information

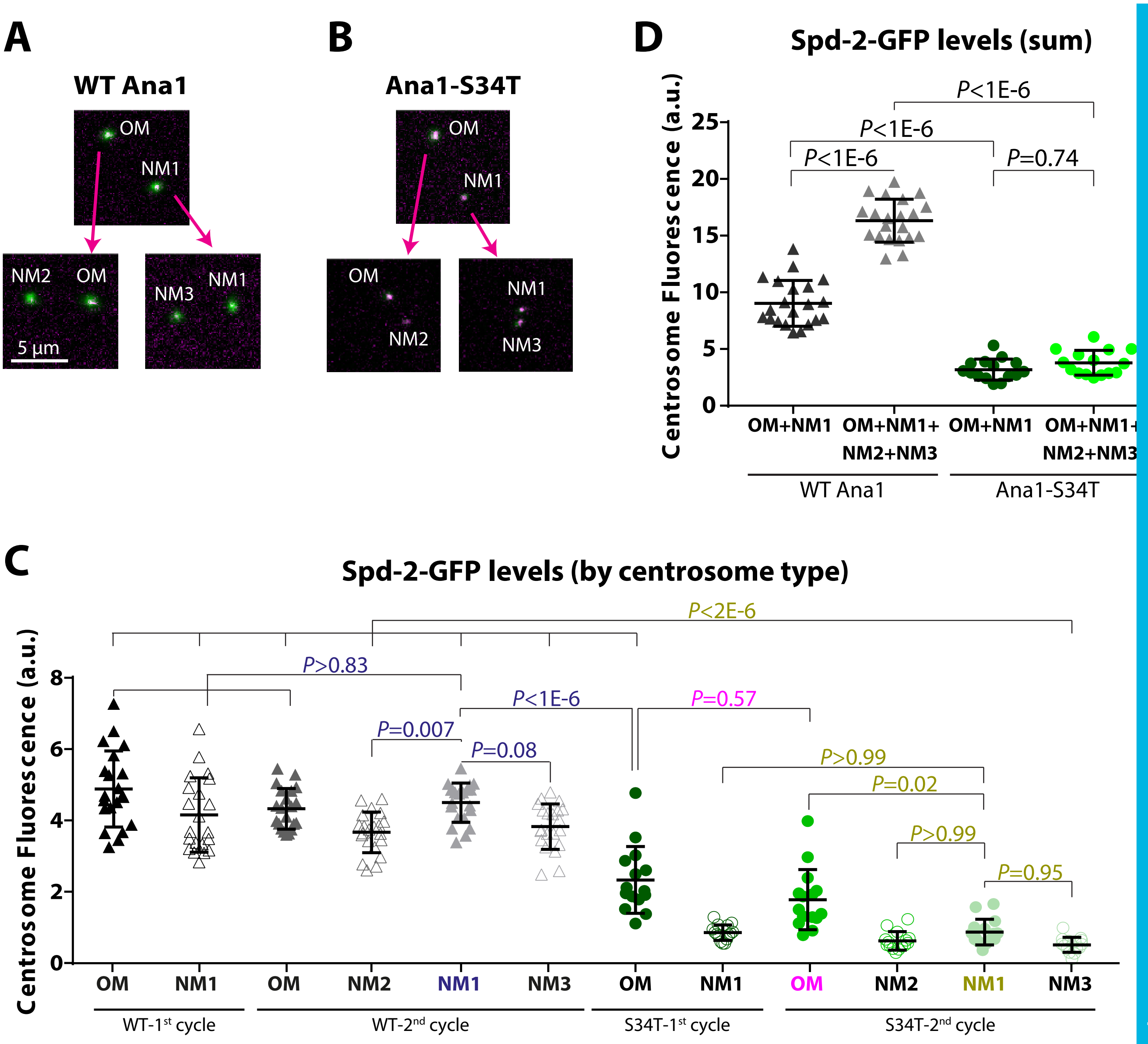

Journal of Cell Science • Supplementary information

Figure S4. Some old mother centrioles can partially bypass the requirement for Ana1 to help recruit Polo to centrioles, and so recruit some of the mitotic scaffold protein Spd-2.

This figure shows a similar pedigree analysis of the behaviour of the mitotic scaffold at OM and NM centrioles to that shown in Figure 6, but here showing the behaviour of the Spd-2 scaffold rather than the Cnn scaffold. **(A,B)** Examples of OM1 and NM1 centrosomes generated at the start of the 1st cycle, and the NM2 and NM3 centrosomes they generated at the end of the 2nd cycle in WT Ana1-mCherry (A) or Ana1-S34T-mCherry (B) (*magenta*) embryos expressing Spd-2-GFP (*green*). **(C)** Graph shows the mean Spd-2-GFP intensity at each centrosome type in WT Ana1-mCherry (*black/grey* triangles) and Ana1-S34T-mCherry (*green* circles) embryos. N = 7 and 5 embryos analysed, respectively; three pairs of centrosomes in the 1st cycle were analysed per embryo, so a total of n = 21 and 15 centrosome pairs for each WT and S34T genotype, respectively. To facilitate visualisation, only the *p*-values corresponding to the most informative statistical comparisons are shown, coloured by the type of centrosome being compared against others: WT NM1 in the 2nd cycle (*navy*), S34T OM in the 2nd cycle (*magenta*), and S34T NM1 in the 2nd cycle (*gold*). **(D)** Graph shows the same data as in (C), but expressed as the average sum of Spd-2-GFP levels for OM+NM1 centrosomes in the first cycle (*dark grey* for WT-rescued embryos, *dark green* for S34T-rescued embryos), and the average sum of Spd-2-GFP levels for OM+NM1+NM2+NM3 centrosomes in the second cycle (*light grey* for WT-rescued embryos, *light green* for S34T-rescued embryos). Error bars represent SD.

Journal of Cell Science • Supplementary information

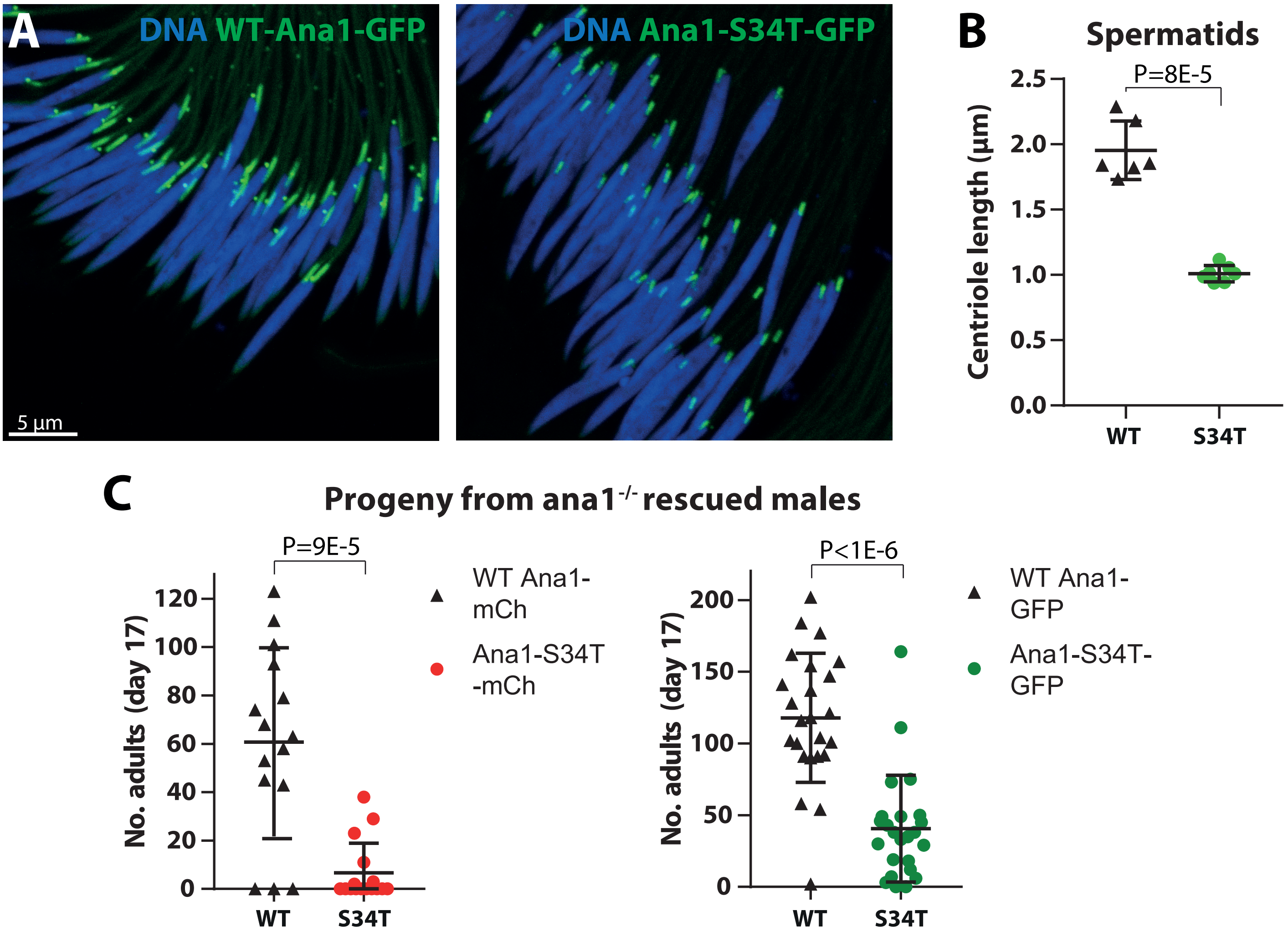
A
DNA WT-Ana1-GFP
DNA Ana1-S34T-GFP
5 µm
B
Spermatids
Centriole length (µm)
P=8E-5
2.5
2.0
1.5
1.0
0.5
0.0
WT
S34T
C
Progeny from ana1-/- rescued males
No. adults (day 17)
P=9E-5
120
100
80
60
40
20
0
WT
S34T
WT Ana1-mCh
Ana1-S34T-mCh
P<1E-6
200
150
100
50
0
WT Ana1-GFP
Ana1-S34T-GFP

Figure S5. *ana1*$^{-/-}$ flies rescued by Ana1-S34T-GFP have shortened basal bodies and exhibit reduced fertility.

(A,B) Micrographs illustrate (A) and graphs quantify (B) how the flagella basal bodies in maturing spermatids are longer in *ana1*$^{-/-}$ mutant spermatids expressing WT Ana1-GFP than Ana1-S34T-GFP (*green*). The tissue was counterstained to reveal the DNA in the sperm heads. The mutant cysts are well organised, and the centrioles/basal bodies are correctly positioned at the base of the nuclei, but they are shorter than normal. (n = 6 and 7 testes respectively, 27-132 centrioles per testis). Error bars represent SD. **(C)** Quantification of the number of flies that emerged 17 days after crossing individual *ana1*$^{-/-}$ mutant males rescued with WT Ana1-mCherry or Ana1-S34T-mCherry (*left*) or WT Ana1-GFP or Ana1-S34T-GFP (*right*) to WT females (n = 15, 16, 24 and 24 individual males, respectively). See Materials and Methods for further details. Error bars represent SD.

Journal of Cell Science • Supplementary information

Protein	Length (aa)	Number of S-S/T motifs	Number of conserved S-S/T motifs	Total (S-S/T motifs/aa)*100 ratio	Conserved (S-S/T motifs/aa) *100 ratio
Sas-4	901	11	7	1.220865705	0.776914539
Asl	994	6	0	0.60362173	0
Cep135	1059	10	2	0.944287063	0.188857413
Ana1	1729	34	14	1.966454598	0.809716599
Spd-2	1146	34	16	2.966841187	1.396160558
Cnn	1148	14	3	1.219512195	0.261324042
Plp	2895	41	14	1.416234888	0.483592401

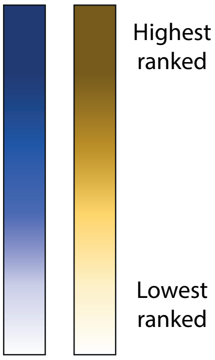

Figure S6: Ana1 and Spd-2 have a relatively high density of potential PBD-binding sites.

Table comparing several different centrosomal proteins on the basis of their protein sequence length (expressed as number of amino acids, aa), total number of potential Polo binding sites (i.e. any S-S/T motif) in their sequence, and number of S-S/T motifs which are conserved amongst at least 11 of 12 *Drosophila* species included in this analysis. The number of total or conserved S-S/T motifs was divided by the total number of amino acids of the protein and multiplied by 100 to calculate the ratio of S-S/T motifs per 100 aa (*blue* column) and the ratio of conserved S-S/T motifs per 100 aa (*yellow* column). The highest four ratios for each column are highlighted as per the colour schemes to the right of the table (darkest tone indicating the highest ratio): Spd-2 and Ana1 have the highest total and conserved S-S/T motifs/aa ratios, followed by Plp (which could not be tested in our assay) and Sas-4 (which has one site that can bind Polo).

Journal of Cell Science • Supplementary information
